# Increased cortical thickness and decreased brain age among special operations veterans with blast TBI after a magnesium-ibogaine protocol

**DOI:** 10.1016/j.isci.2026.115121

**Published:** 2026-02-21

**Authors:** Andrew D. Geoly, John P. Coetzee, Derrick Matthew Buchanan, Wiebke Struckmann, Bora Kim, Malvika Sridhar, Azeezat Azeez, Jennifer I. Lissemore, Kirsten Cherian, Afik Faerman, Jackob N. Keynan, Prakamya Singal, Alaa Shanbour, Igor D. Bandeira, Ian H. Kratter, Maheen M. Adamson, Manish Saggar, Cammie Rolle, Nolan R. Williams

**Affiliations:** 1 Department of Psychiatry and Behavioral Sciences, Stanford University, Palo Alto, CA, USA; 2 Department of Radiology, Stanford University, Palo Alto, CA, USA; 3 Department of Psychiatry, VA Palo Alto Health Care System, Palo Alto, CA, USA; 4 WOMEN CoE, VA Palo Alto Health Care System, Palo Alto, CA, USA; 5 Department of Neurosurgery, Stanford University, Palo Alto, CA, USA; 6 Department of Rehabilitation, VA Palo Alto Health Care System, Palo Alto, CA, USA

**Keywords:** Health sciences, Medicine, Medical specialty, Internal medicine, Neurology

## Abstract

Ibogaine is a psychoactive alkaloid with therapeutic potential that may promote neuroplasticity. Its effects on human brain morphometry are unknown. Thirty Special Operations Forces veterans with prior blast-induced TBI participated in an observational study in which they received ibogaine co-administered with magnesium. Structural MRIs were collected at baseline (*n* = 25), initial post-treatment (*n* = 25), and 1-month post (*n* = 22). Longitudinal analyses assessed cortical thickness, subcortical volume, and predicted brain age (pBA), estimated from T1 scans. pBA was significantly reduced at 1 month relative to baseline (−1.3 years). Cortical thickness analysis revealed post-treatment increases in 11 regions. Subcortical analyses revealed significant volumetric expansion in 8 regions. Magnesium-ibogaine therapy was associated with increased cortical thickness, subcortical expansion, and reduced pBA at 1 month. Although T1s are sensitive to nonstructural changes, the overall direction of effect is consistent with neuroplastic change.

## Highlights

• Magnesium-ibogaine is associated with increased cortical thickness in veterans with blast TBI
• Predicted brain age was reduced by a mean of ∼1.3 years at 1 month post-treatment
• Subcortical volume expansion occurred in cerebellar, forebrain, and diencephalic regions
• Findings compatible with increased structural neuroplasticity after magnesium-ibogaine

## Graphical abstract

**Figure:**
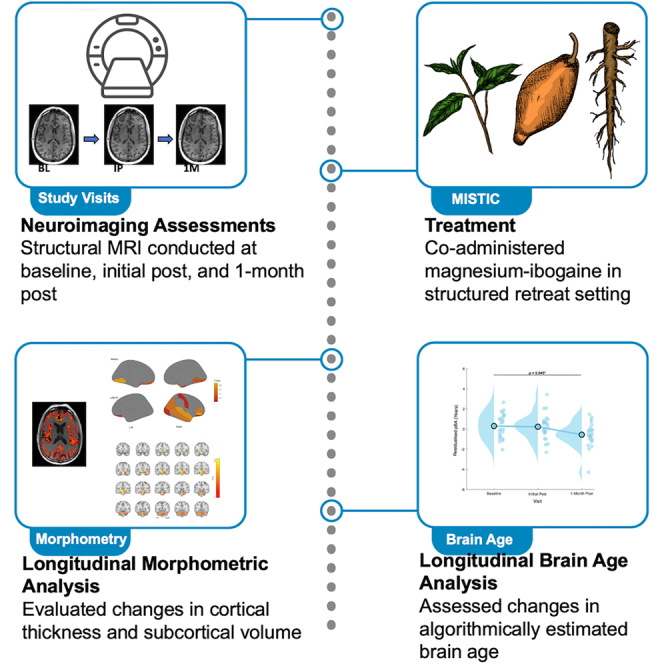

## Introduction

### Traumatic brain injury

Over 50 million individuals per year sustain a traumatic brain injury (TBI) globally, with incidence and prevalence rates increasing over the past three decades.^1^ The leading cause of TBIs incurred by veterans of recent conflicts in Afghanistan and Iraq is blast exposure from improvised explosive devices (IEDs).^2^ Blast-related TBIs have a unique etiology and sequelae commonly seen in current and former military personnel.^3^ TBIs are either focal or diffuse, with focal injury resulting from collision forces acting on the skull, leading to tissue compression at the site of impact (coup) or opposite the impact (contre-coup).^4^ Blast-related TBI, conversely, is a diffuse injury involving widely distributed damage to axons, vascular structures, hypoxic-ischemic injury, edema, and astrocyte scarring.^4^ Blast-related TBIs are typically caused by the detonation of explosive devices, whether intentional, such as in breaching maneuvers,^5^ or unintentional, such as IEDs.^6^ The detonation of these devices produces a supersonic shockwave that can rapidly and transiently increase and then decrease pressure in the cranium and the body within the blast radius.^7^

While many recover from TBI within weeks, approximately 11.4 million Americans are living with at least one long-term TBI-related disability, which can significantly impact their quality of life.^8^ Additionally, many patients with TBI go on to suffer a constellation of long-term health complications, often referred to as persistent post-concussion syndrome (PPCS), a complex, chronic, disabling, and difficult-to-treat condition.^9^^,^^10^^,^^11^ Patients with PPCS may exhibit sleep disturbances,^12^ cognitive deficits related to executive functioning (e.g., decision making/problem solving),^13^ reduced reaction time/processing speed,^14^ impaired attention,^15^ learning,^15^ short and/or long-term memory,^16^ emotional dysregulation, and increased risk for comorbid physical and mental health conditions. Individuals with TBI also have elevated risk for a variety of sequelae, including major depressive disorder,^17^^,^^18^ PTSD,^19^ suicide,^20^ substance abuse,^21^ stroke,^22^ and neurodegenerative diseases, including dementia and Alzheimer’s disease.^23^

There are few effective treatment options for individuals living with TBI-related disabilities.^24^ The complex deficits associated with TBI are rarely fully addressed by any single treatment, and deficits in attention and memory, in particular, may make it difficult for patients with TBI to benefit from conventional first-line treatments, such as cognitive rehabilitation or psychotherapy.^25^ The neurodegenerative pathology following chronic symptoms of TBI can include disrupted white matter integrity and gray matter reductions, such as compromised diffusion and cortical thinning, respectively.^26^ Morphometric changes may also indicate accelerated brain aging,^27^ increasing the risk of developing dementia^23^ as predicted by machine learning algorithms such as brainageR.^28^ With millions of civilians and military veterans suffering daily from TBI, identifying rapid-acting interventions is critically important.

Currently, blast TBI can only be definitively diagnosed postmortem through tissue histology, which is used to identify the presence of interface astroglial scarring at the boundaries between brain parenchyma and fluids, as well as at junctions between gray and white matter,^29^ a feature which also distinguishes blast TBI from chronic traumatic encephalopathy (CTE).^29^ Recent advances in multidimensional magnetic resonance imaging may soon make it possible to definitively diagnose blast TBI in living patients, but until that time, diagnosis will continue to be accomplished primarily through clinical interviews.^30^

### Ibogaine

Ibogaine is a naturally occurring psychoactive compound that has garnered interest as a rapid-acting therapeutic intervention for treating many psychiatric sequelae of TBI suffered by veterans, such as addiction, depression, and anxiety disorders.^31^ Animal studies have demonstrated the neuroplasticity-promoting effect of ibogaine through the transcriptional upregulation of glial cell-derived neurotrophic factor (GDNF),^32^ brain-derived neurotrophic factor (BDNF),^31^ and nerve growth factor.^33^ These effects may promote neural repair at the cellular level.^31^ The repair and replacement of damaged neural tissue may be associated with detectable increases in cortical thickness and volume.^34^^,^^35^

Regarding pharmacology, ibogaine (and its longer lasting metabolite, noribogaine^36^) appears to interact with multiple neurotransmitter systems, including glutamatergic, nicotinic, sigma, mu and kappa opioid, serotonergic, and dopaminergic systems.^37^^,^^38^^,^^39^ While the central mechanism of ibogaine’s effects is not yet confirmed, preclinical studies suggest that antagonism of glutamatergic N-methyl-*d*-aspartate (NMDA) receptors plays a critical role.^38^^,^^40^^,^^41^ Similarly, ibogaine is a noncompetitive inhibitor of the serotonin transporter, which may contribute meaningfully to its antidepressant and anxiolytic effects.^42^^,^^43^ Overall, ibogaine has a set of pharmacological and subjective effects that make it distinct from the more predominantly serotonergic classical psychedelics such as psilocybin.^44^

Despite its potential benefits, ibogaine is not without risk. The scientific literature reports at least 30 fatalities and toxic adverse events associated with ibogaine treatments,^44^ although deaths related to ibogaine tend to be associated with pre-existing medical conditions, overdose, and drug-drug interactions.^35^ Most notably, ibogaine’s potential for causing QT prolongation and the development of Torsades de Pointes (TdP) poses a meaningful clinical challenge.^44^^,^^45^^,^^46^^,^^47^

#### Magnesium-Ibogaine

*Stanford Traumatic Injury to the CNS* (MISTIC) is a therapeutic ibogaine protocol that incorporates the prophylactic co-administration of magnesium in an attempt to reduce potential cardiac-related risks without altering the ibogaine experience. It is worth noting that magnesium can also act as an NMDA antagonist,^48^ and there may therefore be additive antagonistic effects of ibogaine and magnesium on NMDA receptors.^49^ In a recently published first-of-its-kind observational pilot study,^24^ participants saw remarkable clinical improvements in self-reported disability, psychiatric symptom burden, cognitive and neuropsychological performance, and sleep. Although pre-post ibogaine studies in similar populations had been reported before,^50^^,^^51^ this 2024 article^24^ was the first reported study, to our knowledge, on co-administering ibogaine with magnesium. We hypothesized that these improvements would be accompanied by detectable changes in cortical thickness and volume using MRI.

In the present study, we conducted a morphometric analysis of MRI data collected during the aforementioned clinical trial^24^ focused on cortical thickness and parcellated brain volume. We also measured algorithmically predicted brain age by applying the brainageR algorithm^28^ to anatomical T1w MRIs from each visit. This algorithm has been used in several other published studies to characterize the accelerated brain aging often associated with TBI.^27^^,^^52^^,^^53^ Given the profound clinical efficacy reported previously by our group,^24^ our goal was to investigate whether morphometric changes in the brain post-ibogaine infusion accompany the previously reported clinical response in this sample.

The three primary outcome measures being considered were changes from baseline to 1 month posttreatment in cortical thickness, subcortical volume, and predicted brain age. The human cerebral cortex is a highly folded sheet of gray matter (neurons) for which thickness is measured as the distance from the outer boundary (between gray matter and cerebrospinal fluid) to the inner boundary (between the gray matter and white matter),^54^ and this thickness typically ranges from 1 to 4.5 mm, with an overall average of approximately 2.5 mm, with large intraindividual variations.^55^ Cortical thickness is of great interest in normal development and as a marker of normal aging processes, as well as neurodegenerative and psychiatric disorders.^55^ A battery of methods has been developed for measuring cortical thickness from T1 MRI scans.^26^^,^^54^^,^^56^^,^^57^ Subcortical volume refers to the size (measured in cubic mm or cubic cm) of brain structures located ventrally and medially to the cerebral cortex. Subcortical volumes can be derived through the automated segmentation of MRI T1 scans using software such as Freesurfer^58^ or ANTS,^59^ which identify boundaries between these structures and then estimate their volume.^60^ Predicted brain age is an age estimate produced by machine learning models trained on neuroimaging features, typically from structural MRI scans, reflecting the apparent biological age of the brain.^27^^,^^28^^,^^61^ There are a variety of brain age estimation algorithms available. The one used for the current study was brainageR, because of its prior use with TBI populations.^27^^,^^62^

## Results

### Participant characteristics

Participant baseline characteristics are summarized in Table 1. Participants (25 male, mean age 44.5 years, SD = 7.0 years) had a combat exposure scale (CES) score of 29.9 (SD = 5.5), with an average of 65.2 TBIs in their lifetime. Of the 25 participants included, 23 had a mild TBI, 1 had a moderate TBI, and 1 had a moderately severe TBI.^24^ Six participants were unemployed at the time of the study, and 3/6 were unemployed due to disability. Participant characteristics across timepoints are summarized in Table 2.

**Table 1:** Participant characteristics at baseline

Characteristics	Mean (SD)	Demographics	Count (%)
Chron. age in yrs.	44.5 (7.0)	**Race**	
Years of education	15.7 (2.3)	White	21 (84)
CES score	29.9 (5.5)	More than one	3 (12)
BATL total score	12.5 (4.2)	Other	1 (4)
Nr. of TBIs	65.2 (98.6)^a^	**Ethnicity**	
Nr. combat depl.	5.4 (2.4)	Hispanic	1 (4)
**Diagnosis**	**Count (%)**	Non hispanic	24 (96)
PTSD	21 (84)	**Handedness**	
MDD	14 (56)	Right	25 (100)
Anxiety disorder	11 (44)	**TBI severity**	
AUD	12 (48)	1 (mild)	23 (92)
SUD	6 (24)	2 (moderate)	1 (4)
		3 (moderately severe)	1 (4)
		**Suicidality**	
		Past suicidal ideation	17 (68)
		Past suicide attempt	6 (24)
		Past self harm	5 (20)

AUD, alcohol use disorder; BATL, Boston Assessment of Traumatic Brain Injury–Lifetime; CES, combat exposure scale score; Chron. Age, chronological age in years; Nr. combat depl., number of deployed combats.

a Nr. Of TBIs = number of traumatic brain injuries (lifetime). 3/25 participants had blast exposures too numerous to count and hence were unable to report an exact number of TBIs (NrTBIs). Hence, this value was imputed from the original cohort’s (*N* = 30) mean and standard deviation (SD) as mean+5xSD. MDD, major depressive disorder; PTSD, post-traumatic stress disorder; SUD, substance use disorder.

**Table 2:** Participant characteristics across visits

Characteristics, mean (SD)	Baseline (*n* = 25)	Initial Post (*n* = 25)	1 Month (*n* = 22)
pBA in yrs.	39.4 (10.2)	39.0 (10.5)	38.5 (10.4)
Nr. TBIs	66.9 (97.3)	66.9 (97.3)	52.2 (83.8)
Chron. age in yrs,	44.5 (7.0)	44.5 (7.0)	44.9 (7.3)
CES score	29.9 (5.5)	29.9 (5.5)	30.3 (5.7)
WM SNR	15.5 (2.5)	16.2 (2.4)	17.1 (2.0)
GM SNR	11.1 (0.8)	11.2 (0.9)	11.5 (0.7)

CES, combat exposure scale score; Chron. Age, chronological age in years; GM SNR, gray matter signal to noise ratio; pBA, predicted brain age; WM SNR, white matter signal to noise ratio.

### Cortical thickness

We conducted a Wald *Χ*^2^ test of the whole-brain average cortical thickness LME model, which suggested a significant main effect of study visit (*Χ*^2^ = 7.23, *p* = 0.027). Estimated marginal means indicated a subtle increase in average cortical thickness from baseline (2.08 mm) to the initial post-treatment visit (2.13 mm) and 1-month follow-up (2.13 mm). Pairwise contrasts were trend-level after correction (0.05 < p_holm_ < 0.1), consistent with a modest but directionally consistent global pattern of cortical thickening.

**Figure 1:**
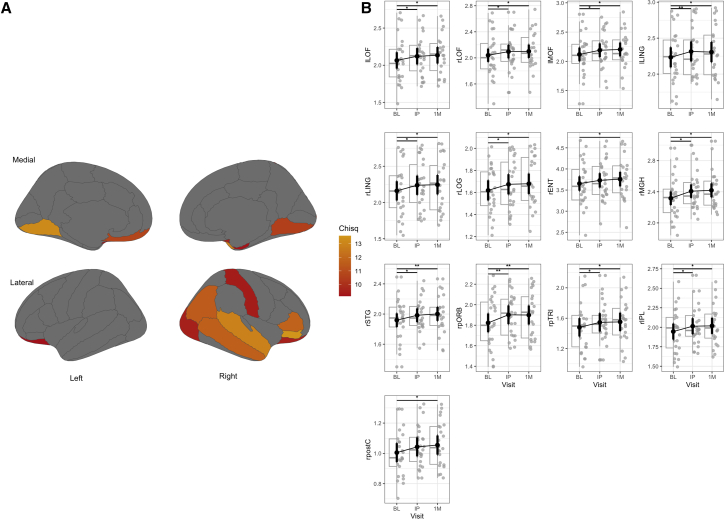
Significant regional changes in cortical thickness following MISTIC (A) Regions with a significant main effect of visit for cortical thickness (surviving FDR correction). The top row indicates the medial view, and the bottom row corresponds to the lateral view of the left and right hemispheres, respectively. The red-to-orange color bar corresponds to the *Χ*^2^ Statistic for the main effect of visit on cortical thickness. Regions illustrated displayed pFDR <0.05. (B) Box-jitter post-hoc pairwise comparisons by visit contrast for regions with significant main effects reveal significant increases in cortical thickness from baseline (BL) to initial post-treatment (IP) and 1-month post (1M) visits. Visit labels are abbreviated for ease of visualization. *Note.* Box-jitter light vertical whiskers represent 1.5 times the interquartile range (IQR) extending from the box edges. Boxes represent medians (solid horizontal lines) and IQR (box edges), solid circles represent model-estimated marginal means, thick error bars represent model-based standard errors, *p*-values are Holm-Bonferroni corrected for three pairwise contrasts with surviving effects (p_holm_ < 0.05) where *p* < 0.05∗, *p* < 0.01∗∗, and *p* < 0.001∗∗∗. For a follow-up normative analysis related to this data please see Tables S9–S11 and Figure S4.

Wald *Χ*^2^ tests of regional LME models revealed a significant (pFDR <0.05) main effect of study visit on cortical thickness in 13 of the 62 examined regions of interest (Figure 1A and Table 3). Subsequent post-hoc pairwise t-tests demonstrated significant (p_holm_ < 0.05) increases in cortical thickness initially following MISTIC therapy relative to the baseline visit in 11 regions (Figure 1B and Table 3). We also observed significant increases in cortical thickness from baseline to 1-month following treatment in the rENT and the rpostC in addition to the aforementioned 11 regions. We found no significant changes between initial post and 1-month post visits, suggesting a likely sustained increase in estimated cortical thickness across the follow-up period. Additional estimated marginal means and post-hoc comparisons can be found in Tables S4 and S5. Overall, there were 11 cortical regions showing significant increases in thickness from baseline to initial post, 13 cortical regions showing significant increases in thickness from baseline to 1 month, and zero regions showing significant increases from initial post to 1 month.

**Table 3:** ANOVA table of regions with significant main effect of visit for cortical thickness (surviving FDR correction)

Region	*Χ*^2^ Statistic	Df	*p*-value	*p*FDR	Initial post - Baseline (mm)	1-month post - Baseline (mm)
**lLING**	13.1865	2	0.0014	0.0325∗	0.0813	0.0727
**lLOF**	9.1448	2	0.0103	0.0493∗	0.0555	0.0687
**lMOF**	10.7307	2	0.0047	0.0405∗	0.0711	0.0848
**rENT**	9.2025	2	0.0100	0.0493∗	0.0749	0.1049
**rIPL**	11.5263	2	0.0031	0.0325∗	0.0688	0.0733
**rLING**	10.5089	2	0.0052	0.0405∗	0.0776	0.0850
**rLOF**	9.5394	2	0.0085	0.0493∗	0.0552	0.0608
**rLOG**	9.5011	2	0.0086	0.0493∗	0.0551	0.0601
**rMGH**	11.7185	2	0.0029	0.0325∗	0.0910	0.1019
**rpORB**	13.5800	2	0.0011	0.0325∗	0.0786	0.0763
**rpostC**	9.4162	2	0.0090	0.0493∗	0.0376	0.0499
**rpTRI**	11.8226	2	0.0027	0.0325∗	0.0656	0.0717
**rSTG**	12.8483	2	0.0016	0.0325∗	0.0651	0.0834

*Χ*^2^ Statistic, Wald chi-square test (Type II). *p*-value: uncorrected *p*-value for the main effect of Visit. pFDR: FDR-corrected *p*-value for 62 regions *p* < 0.05∗, *p* < 0.01∗∗, and *p* < 0.001∗∗∗. lLing, left lingual; lLOF, left lateral orbitofrontal; lMOF, left medial orbitofrontal; rENT, right entorhinal; rIPL, right inferior parietal; rLING, right lingual; rLOF, right lateral orbitofrontal; rLOG, right lateral occipital; rMGH, right middle temporal; rpORB, right pars orbitalis; rpostC, right postcentral; rpTRI, right pars triangularis; rSTG, right superior temporal.

### Normative modeling of cortical thickness

We conducted Wald *Χ*^2^ tests of normative LME models, which revealed a significant main effect of study visit on the aggregate whole brain (*X*^*2*^ = 6.724, *p* = 0.0347) and targeted normative percentile ranks (*X*^*2*^ = 13.302, pFDR = 0.001293). Post-hoc testing revealed significant (p_holm_ < 0.05) increases in targeted normative percentile rank between baseline and initial post-treatment MISTIC (estimated mean difference = +7.49 in relative percentile) and between baseline and 1-month post-MISTIC (Estimated Mean Difference = +8.70 in relative percentile). However, post-hoc testing for the whole brain analysis, we only identified trending increases in percentile rank between baseline and initial post-treatment MISTIC (Estimated Mean Difference = +4.921 in relative percentile) and between baseline and 1-month post-MISTIC (Estimated Mean Difference = +5.546 in relative percentile) (p_holm_ = 0.077). Full ANOVA summary tables, estimated marginal means, pairwise contrasts, and panel figures can be found in Tables S9–S11 and Figure S4, respectively.

### Subcortical volume

**Figure 2:**
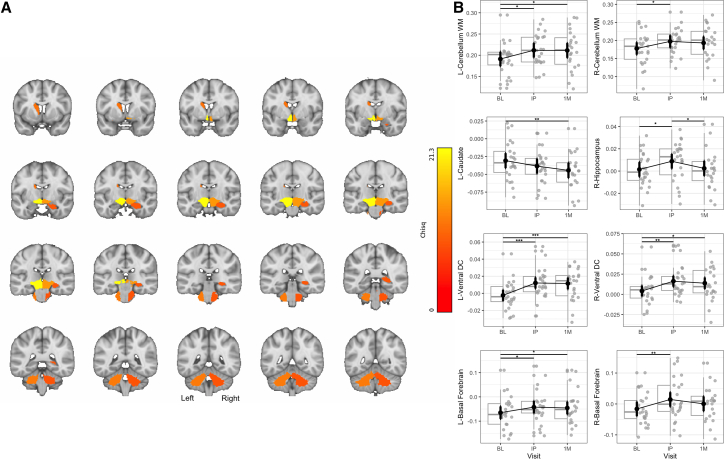
Significant regional changes in subcortical volume following MISTIC (A) Significant main effect of visit for volume (surviving FDR correction) in coronal view. The red-to-orange color bar corresponds to the *Χ*^2^ Statistic for the main effect of visit on volume (log-jacobian determinant). Regions illustrated displayed pFDR <0.05. (B) Box-jitter post-hoc pairwise comparisons by visit contrast reveal significant volumetric changes from baseline (BL) to initial (IP) and 1-month post (1M) visits relative to the study template. For context on these changes, see the discussion section. *Note.* Box-jitter light vertical whiskers represent 1.5 times the interquartile range (IQR) extending from the box edges. Boxes represent medians (solid horizontal lines) and IQR (box edges), solid circles represent model-estimated marginal means, thick error bars represent model-based standard errors, *p* values are Holm-Bonferroni corrected for three pairwise contrasts with surviving effects (p_holm_ < 0.05) where *p* < 0.05∗, *p* < 0.01∗∗, and *p* < 0.001∗∗∗.

We conducted a Wald *Χ*^2^ test of the 28 subcortical LME models, which revealed a significant *p*FDR <0.05∗) main effect of the study visit on the log-jacobian determinant in 8 subcortical regions of interest (Figure 2A). Post-hoc testing revealed significant (p_holm_ < 0.05) volumetric expansion between the baseline and post-treatment MISTIC bilaterally in the cerebellar white matter, basal forebrain, and ventral diencephalon regions, as well as the right hippocampus. Sustained expansions (between baseline and 1-month post-treatment visits) were present bilaterally in the ventral diencephalon, the left cerebellar white matter, and the left basal forebrain, and we identified one volumetric contraction in the left caudate (Figure 2B). We identified significant differences between the post-treatment and 1-month only in the right hippocampus, which reflects a transient observed volumetric expansion that returned to the baseline estimate at 1 month. Additional ANOVA results, estimated marginal means, and post-hoc comparisons can be found in Tables S6–S8. Overall, 7 regions displayed a significant increase in volume from baseline to post-treatment, and 4 regions displayed a significant increase in volume from baseline to 1 month (and 1 region displayed a significant decrease over the same period), while no regions displayed a significant increase in volume from post-treatment to 1 month (and 1 region displayed a significant decrease over the same period).

### Relating morphological and clinical changes

Our exploratory analysis relating changes in cortical thickness, normative percentile estimates, and the log-jacobian determinant (summarized in supplemental information) to changes in the WHODAS-2.0 total score revealed no significant correlations.

### Brain age results

pBA’s produced by the brainageR algorithm ranged from a mean of 39.37 years (SD 10.24 years) at baseline to a mean of 38.54 years (SD 10.40 years) at the 1-month visit. The pBA mean values were slightly lower than chronological ages for this group, which ranged from a mean of 44.52 years (SD 7.01) at baseline to a mean of 44.86 years (SD = 7.32 years). Notably, chronological age, CES score, and number of TBIs were collected once at baseline, while WM SNR and GM SNR were collected from the T1W MRI at each study visit (Tables 1 and 2).

**Figure 3:**
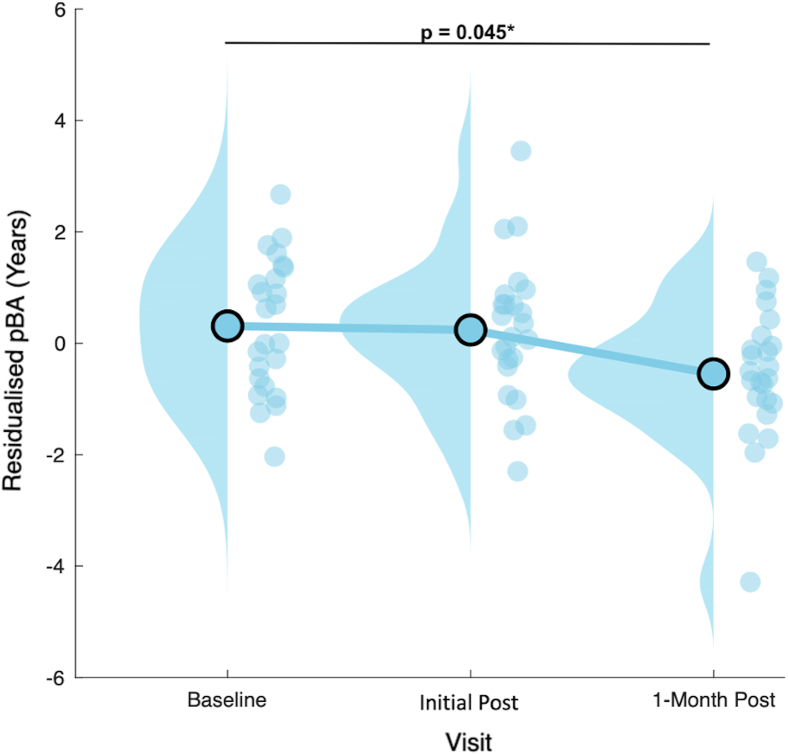
Changes in predicted brain age (pBA) following MISTIC Jitter plots of group-level revisualized pBA in SOV participants at Baseline, Initial-Post, and 1-Month Post MISTIC. *Note.* Solid circles represent model-estimated marginal means; vertical histograms represent the density of estimates by visit; ∗*p*-values are Holm-Bonferroni corrected.

The LME model revealed a significant fixed effect of the variable of interest, study visit, on the dependent variable, pBA (*X*^*2*^ = 6.967, *p* = 0.031∗). Among the covariates, chronological age (*X*^*2*^ = 12.25, *p* < 0.001∗∗∗) and GM SNR (*X*^*2*^ = 4.796, *p* = 0.029∗) had significant effects on pBA (Table 4). Post-hoc pairwise comparisons revealed only a significant difference between the pBA estimated at the baseline visit and the 1-month post visit, giving an estimated marginal mean reduction in pBA of 1.3 years (Table 5; Figure 3).

**Table 4:** ANOVA table of the main effect of study visit for pBA

Parameter	*Χ*^2^ Statistic	Df	*p*-value
Visit	6.967	2	0.031∗
NrTBIs	0.319	1	0.572
Chron. Age	12.254	1	<0.001∗∗∗
CES Score	0.070	1	0.791
WM SNR	0.429	1	0.513
GM SNR	4.796	1	0.029∗

*Χ*^2^ Statistic, Wald chi-square test (Type II). *p*-value: *p* < 0.05∗, *p* < 0.01∗∗, and *p* < 0.001∗∗∗ for the main effect of study visit. pBA, predicted brain age; NrTBIs, number of TBIs (lifetime); Chron. Age, chronological age; CES Score, combat exposure scale score; WM SNR, white matter signal-to-noise ratio; GM SNR, gray matter signal-to-noise ratio.

**Table 5:** Post-hoc pairwise comparisons of pBA

Contrast	Estimate	SE	Df	t-statistic	*p*-value
Initial Post - Baseline	−0.302	0.437	43.1	−0.692	0.493
1-Month Post - Baseline	−1.256	0.496	43.4	−2.531	0.045∗
1-Month Post - Initial Post	−0.954	0.458	43.1	−2.081	0.087

Estimate connotes a difference in model-based estimated marginal means. *p*-values: *p* < 0.05∗, *p* < 0.01∗∗, and *p* < 0.001∗∗∗ are “holm” Bonferroni corrected for 3 pairwise contrasts. Estimate units are years.

### Clinical and neurocognitive relationships

Although potential associations were found between structural brain changes following ibogaine treatment and other study variables, none survived an FDR correction except for the association between pBA_1MnBL_ and HAM-A score at 1-month, normalized to baseline, but a subsequent linear regression did not find a predictive relationship. The relative lack of associations between the changes in brain morphometry and either psychiatric or neurocognitive assessments following MISTIC therapy may be due to inadequate sample size, as the present analysis included only 25 participants (22 when considering the 1-month visit). Previous research has demonstrated that relationships between brain morphometry and neurocognitive performance tend to have small effect sizes and require large samples with wide age ranges for adequately powered analyses.^61^ Moreover, the robust and largely uniform improvement in psychiatric symptoms following MISTIC therapy creates problems for linear statistical analyses (i.e., correlation and regression) that aim to explain the variance of dependent variables. Ergo, while these early patterns of increased cortical thickness, subcortical expansion, and decreases in pBA may be informative mechanistically, larger randomized controlled studies are necessary in order to formally study whether these changes are reproducible and, if so, whether they are related to the clinical effects of MISTIC therapy. For more on the relationship between behavioral and structural outcomes, and how we tested for them, please see the supplemental information.

## Discussion

### Summary

Collectively, these results appear to provide the first assessment on a group level of MISTIC’s effects on gross brain morphometry in humans over the course of a one-month period. Specifically, the present work provides the first preliminary characterization of ibogaine’s effects on brain structure in a cohort of SOV with TBI. This is shown by decreases in pBA as well as longitudinal increases in cortical thickness and subcortical volume in regions that are relevant to the pathophysiology of TBI.^26^^,^^63^^,^^64^^,^^65^^,^^66^^,^^67^

The protocolized administration of ibogaine used in MISTIC^24^ may induce detectable and lasting changes in brain structure in humans. These findings may reflect the growth of gray matter tissue through structural neural plasticity^68^ and support further investigation into the therapeutic potential of ibogaine in the context of TBI, neurodegenerative disease, and treatment-resistant neuropsychiatric conditions.

Interpreting group-level changes in structural morphometry as an indication of therapeutic benefit comes with many challenges. Longitudinal T1 scans may be sensitive to nonstructural changes, such as changes in tissue water content,^69^ and even if increases in cortical thickness, subcortical volumetric expansion, and a decrease in pBA are indicative of structural neuroplastic change, it remains unclear whether these effects are indicative of enhancement/repair or, instead, could be maladaptive. Taken together, the normative analysis findings showing global and region-specific increases in cortical thickness trending toward the 50th percentile, along with reductions in pBA, suggest a pattern of structural change that could be related to the clinical and cognitive benefits described in our prior work, although the present data do not establish a mechanistic link.

### Changes in thickness and volume

The results of this observational pilot study of special operations veterans (SOVs) who underwent MISTIC therapy demonstrated significant group-level increases in cortical thickness initially following MISTIC therapy. Moreover, these regional increases in thickness from baseline were also sustained at the 1-month visit, possibly suggesting durable effects. Areas of increased thickness after treatment were primarily observed in the right hemisphere, especially in the lateral prefrontal (rpORB, rpTRI), temporal (rMGH, rSTG, rENT), and parietal cortices (rIPL, rpostC). We also identified bilateral increases in cortical thickness in the medial occipital lobe (lLING, rLING) as well as the lateral orbitofrontal cortices (lLOF, rLOF). Notably, 10 out of 13 cortical regions showing significant increases in cortical thickness were lateralized to the right hemisphere, whereas a more symmetrical or global distribution of effects would be expected if changes were artifactual. Our findings are supported by previous functional imaging studies on the effects of hallucinogens, reporting increased regional cerebral blood flow and glucose uptake preferentially in the right hemisphere.^70^^,^^71^^,^^72^^,^^73^ While previous studies have found variable patterns of cortical thickness alterations in mild traumatic brain injury (mTBI) cohorts, frontal,^26^^,^^63^^,^^64^^,^^65^ temporal,^26^^,^^63^^,^^64^^,^^66^ and parietal lobe^26^ thinning have been documented when comparing mTBI groups to healthy controls. Interestingly, within the specific context of military blast TBI, cortical thinning patterns have been observed bilaterally in the lateral orbitofrontal cortices, as well as in the right pars orbitalis and pars triangularis.^67^ Consequently, our finding of sustained cortical thickening in these regions is intriguing and possibly suggestive of a putative reversal-like phenomenon of the pathological cortical thinning patterns present in military blast TBI.

Similarly, our log-jacobian determinant analysis revealed statistically significant group-level volumetric expansion following MISTIC therapy, although the timing of these effects was not as uniform as the cortical thickness analysis. Specifically, we identified significant volumetric expansion bilaterally in the cerebellum white matter as well as the basal forebrain, although these effects were only sustained at the 1-month post in the left hemisphere. The collection of heterogeneous structures that make up the basal forebrain area (most notably the basal nucleus of Meynert) has extensive projections to the cortex via both medial and lateral routes. Our analysis also identified a transient expansion in the right hippocampus immediately following MISTIC, as well as a significant volumetric contraction in the left caudate that was only present when comparing baseline and the 1-month post. The caudate is involved in a wide array of functions, including cognitive processes, emotional regulation, motivation and goal-oriented behavior, and motor functions.^74^ Several studies have linked reduced caudate volumes to negative outcomes (e.g., PTSD)^75^; however, while the underlying mechanisms driving this volumetric contraction remain uncertain, and this finding was not consistent with our other results, alternative interpretations of such volumetric reductions are important to consider particularly in the context of improvement of symptoms on various behavioral outcomes and no significant adverse outcomes observed in this sample. One possibility refers to increased synaptic efficiency. Subcortical microstructural abnormalities in the caudate have been associated with PTSD and with impairments in several cognitive measures.^76^ Additionally, greater exposure to adverse childhood experiences (ACEs) may be associated with increased volume in the head of the left caudate.^77^ Previous studies theorized that the elimination of redundant synapses could improve computational efficiency via faster neuronal communication and promote the integration of brain function across diverse regions.^78^^,^^79^^,^^80^ Another potential example of this phenomenon is a recent study showing that mindfulness training may contribute to reductions in caudate gray matter volume and corresponding reductions in positive urgency, defined as the tendency to act impulsively in response to intense positive emotions.^81^ Moreover, volumetric analysis of alcohol-dependent patients has demonstrated that successfully treated patients who would later go on to relapse had elevated bilateral caudate volume at baseline, compared to nonrelapsing patients and healthy controls.^82^ Additionally, Schmidt and colleagues reported increases in caudate volume following 9 years of injectable opioid agonist treatment with diacetylmorphine in 22 patients with opioid use disorder. Notably, the authors report that long-term opioid agonist treatment was associated with the enlargement of the right caudate nucleus, which was related to the duration of opioid use disorder.^83^ As such, the observed reductions in caudate volume may not imply maladaptive changes. Similar observations were made after the pharmacological treatment of patients with obsessive-compulsive disorder.^77^ Finally, we also identified statistically significant group-level expansion bilaterally in the ventral diencephalon (DC) from baseline to immediately following MISTIC therapy, which was also sustained at the 1-month visit. The ventral DC (as defined by the Mindboggle volumetric atlas) encompasses a collection of subthalamic structures,^84^ including the hypothalamus and mammillary bodies. Recent work has implicated this collection of structures in TBI,^85^ Alzheimer’s disease,^86^ late-life depression,^86^ and CTE.^87^

To contextualize our findings of increased cortical thickness post-MISTIC, it is helpful to summarize similar findings from other interventions. For example, electroconvulsive therapy (ECT) is another rapid-acting intervention with high therapeutic efficacy for multiple conditions that is also associated with significant increases in cortical thickness within one week to several months post-ECT.^88^^,^^89^^,^^90^ With ECT, some trials have reported widespread increases in cortical thickness, including some regions that overlap with our findings, such as the inferior parietal gyrus, superior temporal gyrus, postcentral gyrus, right lateral occipital gyrus, and the right superior temporal gyrus. Similarly, although less rapid, pre-post MRI measurements of individuals undergoing antidepressant treatment with sertraline have demonstrated increased cortical thickness in some overlapping areas with our findings, such as medial orbitofrontal, lateral occipital, inferior parietal, superior temporal, and pars triangularis,^91^^,^^92^^,^^93^ and other interventions such as exercise training,^33^ or cognitive training.^94^ A recently published case series demonstrated significant lesion reduction and structural changes following ibogaine treatments in two SOV with TBI who also presented with multiple sclerosis.^95^ Although there were some similarities in the spatial location and directionality of cortical thickness and subcortical volume changes, the small sample size, differences in scanning acquisition parameters, timing of scans, and neurological diagnoses preclude any formal comparisons of the findings to those of the present work. There are also numerous reports of widespread volumetric and cortical thickness enhancements associated with testosterone replacement therapy^96^^,^^97^^,^^98^^,^^99^ and parturition^100^^,^^101^^,^^102^ with time scales as early as four weeks.^103^ It is, therefore, not unprecedented to find that a therapeutic intervention could result in significant increases in cortical thickness or volume. Finally, we note that although we observed significant increases in cortical thickness from baseline to initial post and from baseline to 1 month in many cases, the changes extant at the 1 month timepoint are perhaps more biologically plausible, given that variations at the initial post timepoint may be subject to nonstructural variation that T1 scans sometimes exhibit.

### Change in predicted brain age

The significant reduction in pBA observed here is noteworthy and, importantly, distinct from the demonstrated tendency of certain personal characteristics and behaviors to be protective against accelerated brain aging. For example, female biological sex,^104^^,^^105^ musical expertise,^106^ or extensive meditation experience,^107^ are characteristics and interventions that reduce the anticipated increase in pBA, but do not, of course, reduce the age of the brain itself. Reports in the literature of a medical intervention that reduced pBA are sparse. De Bézenac et al. reported that prior to amygdalohippocampectomy, 48 patients with refractory mesial temporal lobe epilepsy (mTLE) showed an average increased brain age gap (the difference between chronological age and pBA) (BAg) of 7.97 years compared to age-and-sex matched controls (*N* = 37).^108^ Approximately two years following surgery, patients with mTLE (predominantly left-sided) demonstrated an average reduction in BAg of 5.17 years using the brainageR algorithm (same as the present study).^108^ The authors remark that their results support the proposal that mTLE is related to morphologic changes of accelerated aging and that surgical intervention may halt or possibly reverse this process. Studies using different brain-aging approaches from the present work have also demonstrated reductions in pBA. Le et al. reported that administering 200–600 mg of ibuprofen to 20 human participants in a placebo-controlled crossover design resulted in a mean reduction of 1.1 years in predicted brain age using a custom in-house algorithm.^109^ While that study did not identify a mechanism, the authors suggested that it may involve nonselective, reversible inhibition of COX-1, COX-2, and COX-independent pathways. Although no medical intervention was delivered, Luders et al. reported a mean reduction in BAg of 5.4 years between early (1–2 days) and late (4–6 weeks) postpartum stages in 14 healthy postpartum women^101^ using the brain age framework outlined by Franke et al.^110^ While the authors did not identify a significant correlation between the changes in BAg and changes in serum concentrations of estradiol or progesterone, their later work frames the observed reductions in BAg within the context of early-to-late postpartum gray matter increases.^102^

Given that machine learning algorithms such as brainageR function in some ways as a black box, the inner workings of which are obscured from view,^111^ interpreting these results can be challenging. As such, we emphasize caution when interpreting the reduction in pBA as indicating that the brains in question have become younger. More precisely, these results reflect the identification by a machine learning algorithm of anatomical MRI images that more closely resemble brains at an earlier point along the trajectory of healthy aging that it was trained on (James Cole, personal communication). In particular, the increases in cortical thickness reported here likely impacted the age estimates generated by the algorithm, given how consistently cortical thinning is associated with advancing pBA in normally aging individuals.^112^ A neurobiological mechanism that may be involved in both outcomes, increased cortical thickness and reduced brain age, is the upregulation of neurotrophic factors such as BDNF and GDNF, which are associated with ibogaine administration in animal work.^32^^,^^33^

It is worth noting that, although TBI is frequently associated with accelerated pBA,^27^^,^^113^^,^^114^^,^^115^ participants in our study had pBAs that were already lower than their chronological ages, even at baseline. This may be attributable to selection effects; for example, to enter the special forces, these individuals all had to have exceptional physical fitness, which is protective against accelerated brain aging.^116^ It remains to be seen whether this effect would be found in a larger and more diverse cohort over a longer period of time.

Although algorithmic brain age metrics such as predicted brain age are frequently associated with discrete neuroanatomical measures such as cortical thickness and/or hippocampal volume, such metrics do not appear to be reducible to specific neuroanatomical variables. In one dataset including male and female veterans, as well as male and female healthy controls, neuroanatomical variables such as cortical thickness were predictive of brain age metrics in some groups (patients with TBI) but not others (healthy controls).^117^ Additionally, brain age metrics may be more predictive of health trajectory than specific neuroanatomical variables taken by themselves. For example, one study found that brain age metrics were more predictive of the transition to Alzheimer’s disease in an at-risk population than cortical thickness, and another study^118^ found that among patients with Parkinson, brain age metrics predicted the development of MCI even when controlling for cortical thickness. Because algorithmic brain age metrics take a large number of neuroanatomical variables into account, as well as the relationships between them, they may reflect patterns more complex than what any discrete neuroanatomical variable can offer by itself. They have also been of particular interest in the veteran TBI population, where researchers have sought to use such measures to chart the enhanced risk of dementia found in such populations.^27^^,^^52^^,^^53^

### Cerebellar effects

Importantly, as described previously,^24^ all participants experienced transient cerebellar signs during MISTIC therapy, such as mild ataxia and intention tremor. Of note, these incidents were transient and resolved within 24 h following MISTIC therapy completion. Early rat studies reported that high-dose ibogaine administration resulted in cerebellar Purkinje cell degeneration.^119^^,^^120^^,^^121^^,^^122^ These studies contextualize lingering concerns surrounding potential ibogaine-induced neurotoxicity. Although there are considerable limitations when making a cross-species comparison of *ex vivo* tissue histology findings in rats with the present volumetric neuroimaging analysis, we identified significant volumetric expansion (increase in the log-jacobian determinant) bilaterally in the cerebellar white matter following MISTIC therapy, and these changes were not accompanied by volumetric contraction in any cerebellar gray matter regions, which may support an interpretation that these effects were not detrimental, and possibly indicative of remyelination.^123^ Although we did not uncover any evidence of long-standing cerebellar toxicity in the present study, it is important to note that the resolution and specificity of our anatomical T1w MRI (0.9 mm isotropic) in comparison to that of postmortem histological analyses (microns) precludes any formal claims surrounding ibogaine’s neurotoxicity profile in humans, although at least one postmortem neuropathological examination of a patient who used ibogaine on multiple occasions found no evidence of ibogaine-related neurotoxicity.^124^

We reported here on the structural neuroimaging results of an observational study in which thirty SOV, all diagnosed with TBI and multiple comorbidities, underwent MISTIC therapy and experienced substantial improvements in clinical and cognitive scores (as reported in prior work).^24^ We subsequently analyzed MRI data from 25 of those individuals. T1w MRIs were processed using a longitudinal pipeline developed by ANTs^125^ in order to measure changes in cortical thickness and volumetric measures, and using the brainageR^62^ algorithm in order to obtain pBAs. MISTIC therapy was associated with significant pre-to-post increases in cortical thickness, primarily at the 1-month visit, contrasted with baseline, and with significant pre-to-post reductions in pBA, also at the 1-month visit, contrasted with baseline. These changes in brain structure may, at least in part, be associated with the therapeutic action of ibogaine, although this remains to be established.

### Limitations of the study

This study had a number of limitations. First, the lack of a control condition makes it difficult to evaluate whether the changes observed here were in some way due to a placebo effect. While it is unlikely that placebo effects could lead to structural brain changes, they could lead to the adoption of healthier lifestyles or reduced stress, which in turn could lead to structural brain changes.^126^^,^^127^^,^^128^^,^^129^ The ubiquity of such effects makes the interpretation of neuroimaging results something that should be engaged in with caution.^130^ Second, biological samples (i.e., blood, urine, and saliva) were not collected for this study, meaning we have little information about whether circulating levels of potentially relevant neurotrophic factors were changed by the treatment, and so we can only speculate about the potential role of those substances in producing the effects observed here. Of course, the limited sample size impairs our ability to detect small to moderate effects in this sample, a drawback that we hope can be addressed in future studies.

Methodologically, we cannot exclude the possibility that our results might differ due to alternative image acquisition parameters, processing, or analytic approaches, although our comparison of the ANTs and Freesurfer pipelines was supportive of our current approach. Similarly, it is worthwhile to note that the formal diagnosis of blast-related TBI is based on clinical history and/or personal recollection, and verification is not currently possible with MRI alone and would require postmortem tissue analysis to identify astrocyte scarring.^29^ Additionally, for the normative modeling analysis, we were unable to apply a more sophisticated site-harmonization technique due to a lack of healthy control participants in our sample, and the dual differences of scanners and TBI cannot necessarily be untangled. However, while tentative and exploratory in nature, we think this is still potentially a valuable analysis for laying the groundwork for future studies to compare ibogaine effects to a normative sample. Finally, while our sample may be representative of highly trained male military SOVs with multiple blast injuries and superior physical fitness who are seeking ibogaine therapy, it is otherwise lacking in generalizability. Many questions remain about the effects of MISTIC and of ibogaine, more generally, on the human brain. Future studies will need to include more diverse patient samples to facilitate a broader understanding of the effects of ibogaine therapy. A randomized controlled trial of sufficient size and quality could determine whether the brain changes observed here result from pharmacology, expectation, the therapeutic environment, or, in the most likely case, all three.

The predicted brain age of the participants was, on average, younger than their chronological age, despite this cohort all suffering from multiple TBIs, which are typically associated with predicted brain ages that are higher than the reported chronological age. We do not know what the reason for this was, although two candidate explanations are suggested here. First, participants in this study were all former SOF members with high levels of physical training, and who also mostly continued to engage in high levels of physical activity on a regular basis. Good cardiovascular health has been shown to be associated with lower predicted brain age.^131^ Second, this may be due to properties of the particular MRI that was used to collect images for this study, as we have observed similarly young predicted brain ages for participants in other studies we have conducted using this MRI. These factors should be considered in any future replication.

## Resource availability

### Lead contact

Requests for further information and resources should be directed to and will be fulfilled by the lead contact, John Coetzee (jpcoetzee@stanford.edu).

### Materials availability

This study did not generate new biological materials.

### Data and code availability

• Data: The deidentified human participant data reported in this study cannot be deposited in a public repository because they contain sensitive clinical and neuroimaging data from a vulnerable veteran population and are subject to Institutional Review Board-mandated controlled access. To request access, please contact the Stanford University Institutional Review Board and the study contact (John Coetzee, jpcoetzee@stanford.edu) with a research proposal and data security plan consistent with Stanford data-sharing requirements. The datasets used for normative modeling can be found here: https://github.com/ntustison/PaperANTsX/tree/master/Data.
• Code: This article does not report original code.
• Any additional information required to reanalyze the data reported in this article is available from the study contact upon request.

## Acknowledgments

We would like to acknowledge the financial support of this project by Steve and Genevieve Jurvetson, as well as by the Pritzker Neuropsychiatric Disorders Research Consortium. We also acknowledge the role of VETS in recruiting participants and the Ambio Clinic, which provided the treatment. We also acknowledge Rachel M. Rapier for her assistance in preparing the funding proposal, and Saron Hunegnaw and Mackenzie Mattos for their role in collecting data, and 10.13039/100005492Stanford University for providing institutional support and facilities. Finally, we would like to acknowledge the contributions of our study participants and their families. Dr. Nolan Williams passed away on October 8, 2025. All other authors affirm that Dr. Williams made significant contributions to the conception, design, and interpretation of the work and that this submission is consistent with his intentions. This work is dedicated to his memory.

## Author contributions

Investigation, A.G., J.C., D.M.B., W.S., B.K., M.S., A.A., J.L., K.C., A.F., J.N., P.S., A.S., I.B., I.K., and N.W.; formal analysis, A.G. and J.C.; writing – original draft, A.G., J.C., W.S., D.M.B., and B.K.; conceptualization, funding acquisition, and project administration, N.W., supervision, N.W., M.S., C.R., and M.A.; writing – review and editing, A.G., J.C., D.M.B., W.S., B.K., M.S., J.L., K.C., A.F., J.N., I.K., N.W., C.R., M.S., and M.A. All co-authors have read and approved the final version of the article.

## Declaration of interests

Dr. Williams is a named inventor on Stanford-owned intellectual property relating to magnesium-ibogaine. He has served on scientific advisory boards for Soneira, Salma, Otsuka, NeuraWell, Magnus Medical, and Sooma as a paid advisor. He has held equity/stock options in Soneira, Salma, Magnus Medical, NeuraWell, and Sooma.

Dr. Kratter is named inventor on Stanford-owned intellectual property relating to magnesium-ibogaine; he currently receives a salary from and has equity/stock options in Soneira.

Dr. Coetzee and Mr. Geoly are named inventors on Stanford-owned intellectual property relating to magnesium-ibogaine.

Dr. Adamson has served as a brain injury advisor for Soneira.

All other investigators declare no competing interests.

To mitigate any potential bias of the aforementioned competing interests, Dr. Williams recused himself from any analytic roles or article revision. Dr. Saggar and Dr. Rolle, who are both unconflicted senior investigators, were assigned the role of supervision of data analyses and article revision.

## STAR★Methods

### Key resources table

**Table:** 

REAGENT or RESOURCE	SOURCE	IDENTIFIER
**Software and algorithms**		

Advanced Normalization Tools (ANTs)	Penn Image Computing and Science Lab (PICSL)	https://github.com/ANTsX
R 4.2.0	The R Project for Statistical Computing	https://cran.r-project.org/bin/windows/base/old/4.2.0/
Freesurfer	Harvard University	https://surfer.nmr.mgh.harvard.edu/fswiki/DownloadAndInstall
brainageR	James Cole, PhD	https://github.com/james-cole/brainageR

### Experimental model and study participant details

#### Participants

Thirty male SOVs completed the main clinical study.^24^ Informed consent was obtained prior to enrollment in the study. Due to variations in structural image quality and incomplete MRI acquisition (see MRI quality and data inclusion details below), our final cohort for the present analyses comprises 25 SOVs with a diagnosis of mild to moderate TBI. The imaging analysis was conducted on this cohort at three distinct time points: baseline, initial post-treatment (four to five∼7 days after baseline), and one-month post-treatment. We were not able to assess the effect of sex because all participants were male.

All participants were required to possess the ability to read, understand, and provide written, dated informed consent. Only US citizens between the ages of 18–70 years with a history of head trauma, combat, or blast exposure who had voluntarily enrolled in a tabernanthe iboga exposure retreat at a clinic in Mexico were eligible to participate.^24^

#### Study design

We conducted an observational pilot study to determine safety and structural brain correlates of the MISTIC protocol in SOVs with chronic TBI-related disability and complex comorbid psychiatric symptoms. All research procedures were approved by the Stanford Institutional Review Board (IRB), under Stanford IRB-54095. Participants arrived at Stanford University two to three days before their treatment. During this period, they underwent in-person assessments encompassing self-report measures, clinical and neuropsychological evaluations conducted by a trained neuropsychologist, and MR imaging. Following the evaluations, these 25 participants traveled to the clinic, where they participated in a healing retreat in which they underwent MISTIC treatment, involving approximately 12.05 (SD 1.44) mg/kg of oral ibogaine after an intravenous infusion of 1 mg of magnesium sulfate, both prior to and after ibogaine ingestion.^24^ Subsequently, participants returned to Stanford for a follow-up evaluation four to five days post-treatment, with an additional assessment conducted one month later.^43^ Details on study procedures can be found in a prior manuscript from our group.^24^

### Method details

#### Clinical and neuropsychological assessments

Measures reported in the current manuscript include the Combat Exposure Scale (CES), the Boston Assessment of Traumatic Brain Injury Lifetime (BAT-L), the Ohio State University TBI Identification Method - Short Form (OSU-TBI—Short Form), and the WHO Disability Assessment Schedule 2.0 (WHODAS-2.0). For a complete list of measures administered as part of the original study please see prior work from our group.^24^

#### Behavioral measures administered

A number of clinical and cognitive assessments were performed during this study. For a detailed list please refer to either the supplement of this paper or the prior manuscript from our group.^24^ For the purpose of this work, we examined relationships between longitudinal morphometric changes and improvements in the WHODAS-2.0,^132^ which was the primary clinical outcome of the study.

#### MRI acquisition parameters

A 3 Tesla GE Discovery MR750 scanner with a 32-channel head-neck imaging coil was used to acquire MRI Scans at the Center for Cognitive and Neurobiological Imaging at Stanford University. All participants were screened for MRI safety before scanning procedures. Whole brain structural images were collected using GE’s BRAVO sequence (3D, T1-weighted, FOV = 256 × 256mm; matrix = 256x256 voxel; TR = 6.39 ms, TE = 2.62 ms, slice thickness = 0.9 mm, flip angle = 12°). For each participant, MRI data were acquired at approximately the same time of day across all visits, with an average time difference of 1.5 h (±1.1 h) between visits. All participants were instructed to keep their heads still during the scan. Head motion was restricted using memory foam and inflatable padding. Additionally, participants’ motion was monitored using in-scanner video cameras.

#### MRI data quality and inclusion

Of the 30 participants in the study, usable data from 25 participants’ were included at the baseline and initial post-treatment visits, and 22 of these participants had available data for the 1-month post-treatment (this scan was not collected for all participants). Gray matter (GM) and white matter (WM) signal-to-noise ratio (SNR) estimates were calculated on the raw T1w MRI data using MRIQC.^133^ Because there are no explicit thresholds set for tissue-specific SNR by the authors, we leveraged their publicly available outputs of T1w MRI data from OSF,^134^ which yielded means and standard deviations of 10.52 (2.54) for GM SNR and 17.11 (5.45) for WM SNR. SNR from our data were consistent with these estimates (Table 2) over time.

### Quantification and statistical analysis

#### Cortical thickness measurements with ANTs

For the present analysis, we derived cortical thickness measures for our participants with the ANTs longitudinal cortical thickness pipeline^56^^,^^57^ using *antsLongitudinalCorticalThickness.sh.* The brain template, brain extraction probability mask, tissue segmentation priors, and brain extraction registration mask were derived from the OASIS template.^135^ Briefly, the longitudinal cortical thickness pipeline performs cortical thickness estimation for a longitudinal image series for a single subject by 1) creating an unbiased single-subject template (SST) from all time point images, 2) applying the ANTs cross-sectional cortical thickness pipeline *antsCorticalThickness.sh* to the SST with group template and priors as input, 3) creating of the SST tissue prior probability maps, 4) rigidly transforming each individual time point to the SST, 5) applying the ANTs cross-sectional cortical thickness pipeline to each individual time-point image with the SST as a reference and finally 6) Joint label fusion using the Mindboggle OASIS-TRT-20 DKT 31 atlas labels^125^ to determine cortical ROIs for statistical analysis.

As such, a cortical thickness map for each study visit was output in the SST space for visual quality assurance, along with corresponding DKT 31 atlas ROIs and the estimated total intracranial volume (eTIV) of the SST for each participant. Regional mean cortical thickness estimates were derived using *ImageIntensityStatistics,* and 62 (31L | 31R) cortical ROIs were evaluated statistically.

The ANTs pipeline has been shown to perform exceptionally well for registration^125^ as well as cortical thickness measurement in terms of minimal failure rate, higher reproducibility, and improved predictive performance in thousands of images, even compared to the state-of-the-art.^56^ The ANTs cortical thickness pipeline has also been implemented in morphometric studies of TBI,^61^^,^^136^ Alzheimer’s disease,^137^^,^^138^ depression,^139^^,^^140^ and temporal lobe epilepsy.^141^ As both methods are frequently used in morphometric studies, we also employed the Freesurfer (7.4.0) longitudinal processing pipeline in parallel, and trained raters performed visual inspection and subsequent manual longitudinal editing steps to correct skull-stripping, tissue (WM, Pial) boundary, and atlas labeling errors. DKT 31 atlas ROI cortical thickness estimates were extracted for each participant by study visit. As pipeline evaluation metrics, we performed test-retest reliability and unidimensional reliability analyses for cortical thickness estimates across regions between the two pipelines (see supplemental information for details). Those analyses suggested the superiority of the ANTs pipeline compared to FreeSurfer with respect to our specific dataset and informed the decision to conduct the formal analysis using ANTs.

#### Volumetric measurements with ANTs

As part of the ANTs longitudinal cortical thickness pipeline,^142^ log-jacobian determinant maps are also output for each time point in the SST space. Each value in the log-jacobian map quantifies the relative expansion or contraction of the time point image relative to the (OASIS) study template wherein negative values indicate the time point image < study template and positive values indicate the time point image > study template. We evaluated volumetric statistics in 28 subcortical and cerebellar ROIs from the Mindboggle volumetric atlas using *ImageIntensityStatistics* similar to cortical thickness measurements yielding regional mean log-jacobian determinant estimates.^142^^,^^143^^,^^144^

#### Statistical analysis -- repeated measures

Before evaluating regional effects, we first assessed longitudinal change in whole-brain average cortical thickness using a linear mixed-effects model with the same covariates described below (Visit, age, CES, nrTBIs, eTIV, and a random intercept). This provided an overall measure of global cortical change across visits.

To evaluate longitudinal changes in cortical thickness and volume across the 62|28 cortical |subcortical ROIs, we employed linear mixed effects models in R (4.2.0) using the *lmer* function from the *lme4* package. Consistent with the modeling used in our previous work,^24^ for each ROI, a linear mixed effect model was constructed to determine the main effect of the study visit while covarying for chronological age, combat exposure scale (CES), the number of TBIs (nrTBIs), and the eTIV with a random intercept, and using restricted maximum likelihood (REML).

1) R_i_ ∼ Visit + Age + CES + nrTBIs + eTIV + (1 | Subject)

For each region, adherence to model assumptions was examined qualitatively by evaluating the QQ-plots and histograms of the model residuals. Additionally, we formally tested the fit of model assumptions by computing the skewness of residuals, the percentage of residuals that fell within 2 standard deviations of the mean, and by performing a Kolmogerov-Smirnov test of residuals. Model assumptions were met across the 62 ROI thickness and 28 ROI volume measures 62|28. We note here that the thickness and volume ROIs differed in number because we chose to examine subcortical and cerebellar volumes (28) separately from cortical thickness (62) regions.

Visit main effects were then tested formally using the *Anova* function from the *car* package in R to estimate a Wald Chi-Square statistic from a Type-II test (assuming no interactions). To account for multiple comparisons across 62|28 tests, all main effect *p*-values were adjusted using an FDR correction for thickness and volumetric analyses, respectively.^145^

Where surviving main effects were found, estimated marginal means were derived for each visit (*lsmeans* function from the *emmeans* package), and pairwise comparisons were carried out utilizing a “Holm” Bonferroni correction for 3 pairwise contrasts [baseline - initial -post, baseline - 1-month post, initial-post - 1-month post] defined with Kenward-Roger degrees of freedom.

#### Normative modeling of cortical thickness

To evaluate whether changes in cortical thickness were indicative of a turn toward “health,” we utilized preprocessed DKT-31 labeled cortical thickness data from the IXI (*n* = 246), Kirby (*n* = 21), Oasis (*n* = 152), NKI (*n* = 45) and SRPB (*n* = 532) datasets. These data were made publicly available, all participants were processed through the ANTs cortical thickness pipeline for a single time point, and have undergone rigorous quality control procedures to ensure usability. The data were further restricted to include only male participants without a present or past psychiatric or neurological diagnosis. In total, 996 male healthy control comparison participants were included in normative model creation. To minimize cohort effects, we created a linear regression model for each cohort, independently regressing out age and eTIV effects across cortical thickness measures using the *lm* function from the *stats* package in R as follows.

1. R_i_ ∼ Age + eTIV

We then calculated standardized residuals for each regional model in each cohort using the *rstandard* function from the *stats* package in R.

To create a normative estimate for each of our participants at each study visit, we computed a *Z* score for each of the 62 DKT regions based on the computed regional standardized residual values. We then utilized these regional Z-scores to compute regional normative percentiles for each of our participants at each time point using the *normcdf* function in MATLAB. We then computed a Whole Brain (mean) percentile across regions. Additionally, for the 13 regions that were identified as significant from the first analysis (Figure 1 and Table 1), we also computed a Targeted (mean) percentile rank.

$$Z_{\text{region}}=\frac{\text{resid}\left({\text{CT}}_{\text{region}}\right)-\mu_{\text{norm}\left(\text{resid}\left(\text{region}\right)\right)}}{\sigma_{\text{norm}\left(\text{resid}\left(\text{region}\right)\right)}}$$

Percentile_region_ = *normcdf*(Z_region_)

$${\text{Percentile}}_{\text{Whole}\;\text{Brain}}=\frac{\sum {\text{Percentile}}_{\text{region}}}{N_{\text{regions}}}$$

Finally, we employed linear mixed effects models to evaluate the main effect of visit on Whole Brain and Targeted Percentiles. Of note, because standardized residuals used to compute the normative percentile scores accounted for Age and eTIV, those variables were not included in the mixed effects modeling.

1. Percentile_Whole Brain_ ∼ Visit + CES + nrTBIs + (1 | Subject)
2. Percentile_Targeted_ ∼ Visit + CES + nrTBIs + (1 | Subject)

Visit main effects were then tested formally using the *Anova* function from the *car* package in R to estimate a Wald Chi-Square statistic from a Type-II test (assuming no interactions). Where surviving main effects were found, estimated marginal means were derived for each visit (*lsmeans* function from the *emmeans* package) and pairwise comparisons were carried out utilizing a “Holm” Bonferroni correction for 3 pairwise contrasts [baseline - initial-post, baseline - 1-month post, initial-post - 1-month post] defined with Kenward-Roger degrees of freedom.

Residualised estimates for each of the 62 DKT regions were then utilized to compute normative percentiles, and we computed aggregate (mean) whole brain (62 regions) and targeted (13 regions detected previously) percentile ranks for each participant at each study visit.

We used similar linear mixed effects modeling and subsequent Wald *Χ*^2^ tests to interrogate changes in the whole brain and targeted normative percentile ranks across study visits, except that age and eTIV (which were previously accounted for) were not included. Where surviving main effects were found, estimated marginal means were derived for each visit and we carried out pairwise comparisons similarly to the previous repeated-measures cortical thickness analysis.

#### Statistical analysis, relating morphological and clinical changes

Where significant regional main effects were detected, we evaluated exploratory Spearman’s ***ρ*** correlations between the raw changes in regional estimates and the changes in WHODAS-2.0 total score [initial -post - baseline, 1-month post - baseline]. Statistical significance was determined as *p* < 0.05 following an FDR correction for multiple comparisons.

#### Brain age algorithm

Predicted brain age (pBA) values were calculated from T1-weighted (T1W) MRI scans using brainageR. A full and detailed description of the development, testing, and validation of this algorithm can be found in prior work.^62^ Values for pBA were obtained from this machine-learning analysis of neuroimaging data for all participants.

We then used the resulting pBA values as the dependent variable in a linear mixed effects (LME) model constructed and evaluated in the same manner as was done for the morphometric analyses. Additional fixed effects covariates included the white matter and gray matter signal-to-noise ratio (SNR) of the T1w MRI scan (these last two were included on the basis of personal communication with the brainageR algorithm author). Correlational analyses were conducted to identify associations between changes in pBA and changes in behavioral outcomes (see supplemental information).
