# Supplementary material for: Increased cortical thickness and decreased brain age among special operations veterans with blast TBI after a magnesium-ibogaine protocol
Source: iScience. 2026 Feb 21;29(3):115121. doi: 10.1016/j.isci.2026.115121 (PMC13010113; doi:10.1016/j.isci.2026.115121)
Supplement: Document S1. Figures S1–S4 and Tables S1–S12 [file mmc1.pdf]

iScience, Volume *29*

Supplemental information

Increased cortical thickness and decreased brain age among special operations veterans with blast TBI after a magnesium-ibogaine protocol

Andrew D. Geoly, John P. Coetzee, Derrick Matthew Buchanan, Wiebke Struckmann, Bora Kim, Malvika Sridhar, Azeezat Azeez, Jennifer I. Lissemore, Kirsten Cherian, Afik Faerman, Jackob N. Keynan, Prakamya Singal, Alaa Shanbour, Igor D. Bandeira, Ian H. Kratter, Maheen M. Adamson, Manish Saggar, Cammie Rolle, and Nolan R. Williams

Supplementary Materials

Supplementary Table 1. Bayesian unidimensional reliability statistics.

Estimate	McDonald's ω	Inter-item Correlation
**ANTs SST Pipeline**		
Posterior Mean	0.9700	0.4920
95% CI (lower bound)	0.9630	0.41408
95% CI (upper bound)	0.9769	0.5684
R-hat	0.99915	0.999895
**FS Long Pipeline**		
Posterior Mean	0.8596	0.31696
95% CI (lower bound)	0.82317	0.251735
95% CI (upper bound)	0.89608	0.38222
R-hat	1.0002	1.0011

CI = confidence interval.

We utilized the JASP (0.17.1) Bayesian Unidimensional Reliability module to assess the pipelines' ability to consistently measure the unidimensional construct of cortical thickness [MCMC parameters: 1000 samples, 50 burn-in samples, thinning factor = 1, number of chains = 3)]. We computed a global McDonald's ω and the mean item-to-rest correlation for each pipeline model. Additionally, we also computed region-wise McDonald's ω (if item dropped) and region-wise item-to-rest correlations for each pipeline to identify specific regions where reliability may be different between pipelines. Of note, both models performed with R-hat < 1.01. Our results (Supplementary Table 1) indicate that the ANTs longitudinal SST pipeline performed with superior unidimensional reliability (global McDonald's ω = 0.97, Inter-item correlation = 0.492) as compared to the Freesurfer longitudinal pipeline (global McDonald's ω = 0.86, Inter-item correlation = 0.317).

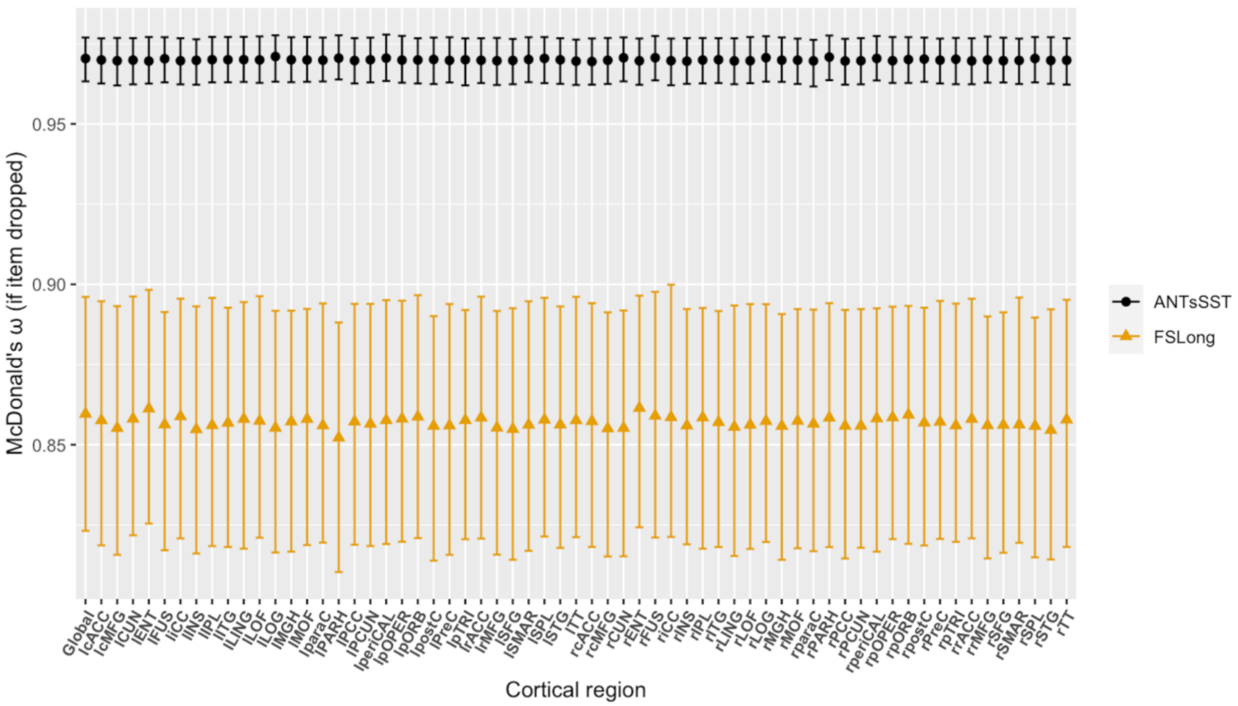

Supplementary Figure 1. Global and regional McDonald's ω between the ANTs longitudinal SST (ANTsSST) and Freesurfer Longitudinal (FSLong) pipelines. Dots and triangles represent posterior means while error bars indicate 95% confidence intervals. *Note*: "Global" estimates connote the overall McDonald's ω and regional estimates connote ω if the item were dropped.

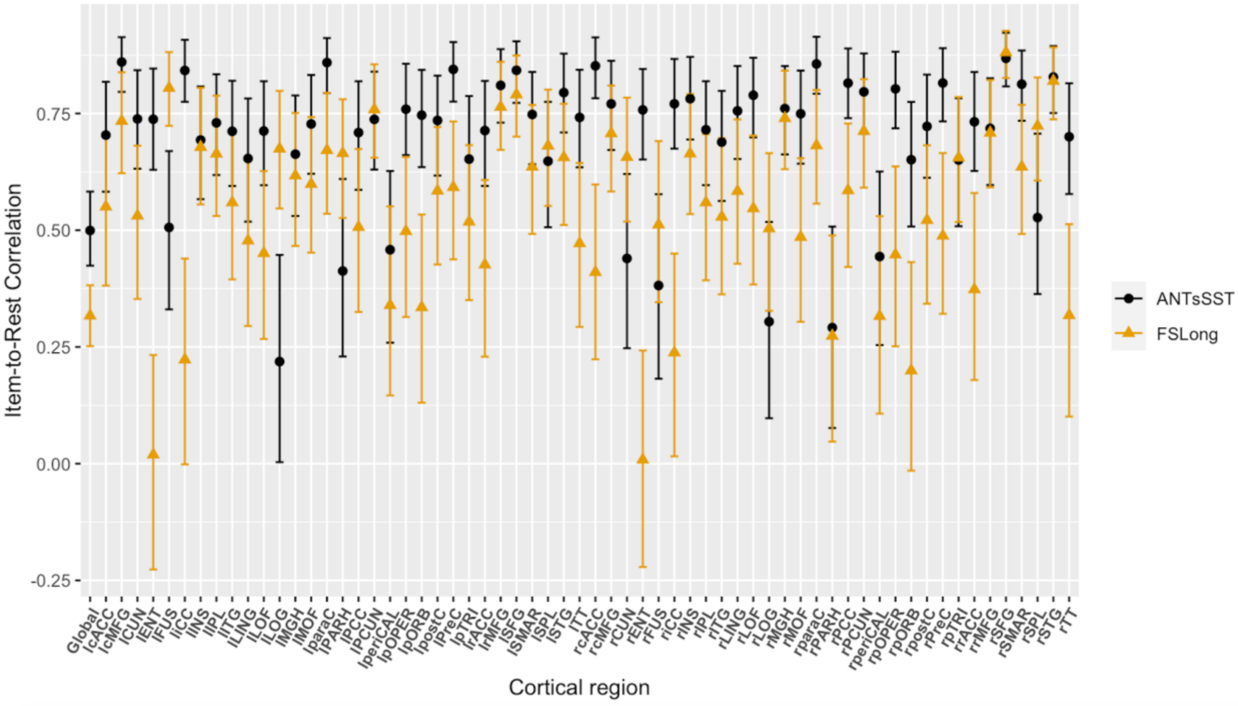

Supplementary Figure 2. Global and regional item-to-rest correlation between the ANTs longitudinal SST (ANTsSST) and Freesurfer Longitudinal (FSLong) pipelines. Dots and triangles represent posterior means while error bars indicate 95% confidence intervals. *Note*: "Global" estimates connote the overall inter-item correlation and regional estimates connote regional item-to-rest correlations.

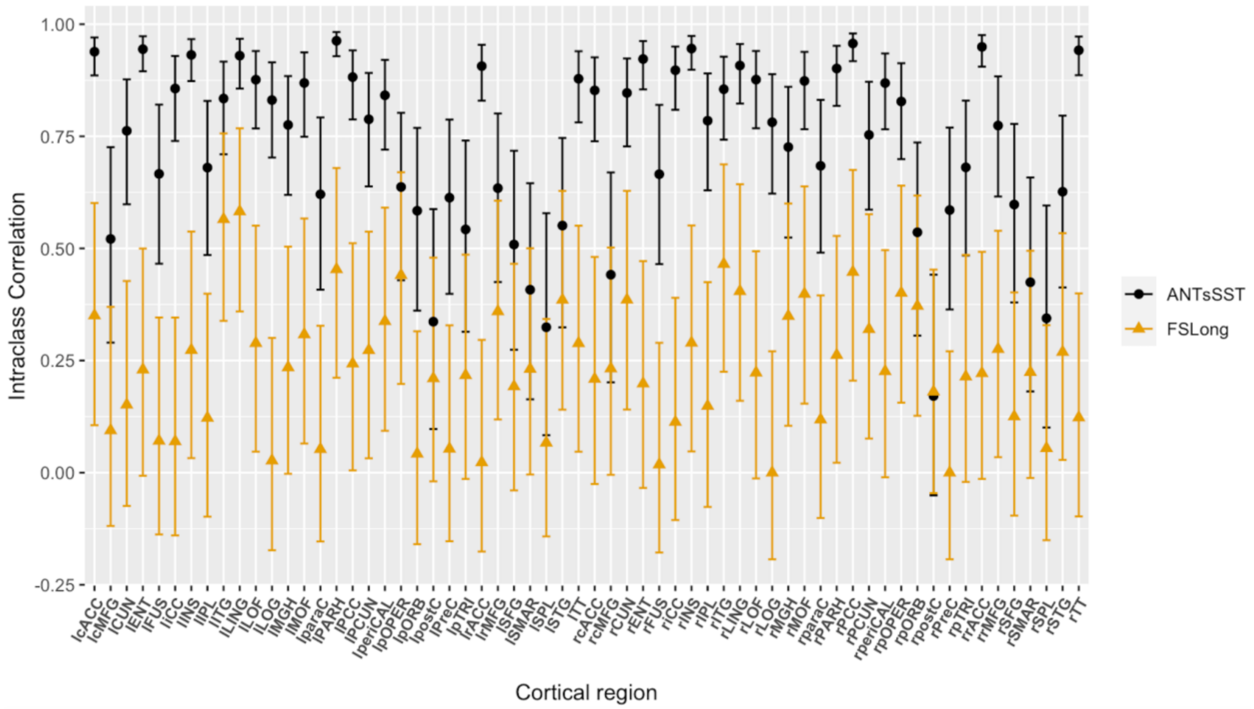

Supplementary Figure 3. To compare the test-retest reliability of each pipeline, we also computed the intra-class correlation coefficient ("ICC(2,1)" notation of Shrout and Fleiss, 1979) for each region (Supplementary Figure 3) with the ANTsSST (Mean ICC = 0.733, Median ICC = 0.783) pipeline outperforming the Freesurfer Longitudinal pipeline (Mean ICC = 0.234, Median ICC = 0.228). Regional ICC between the ANTs longitudinal SST (ANTsSST) and Freesurfer Longitudinal (FSLong) pipelines. Dots and triangles represent regional estimates while error bars indicate 95% confidence intervals.

Repeated Measures Model Fits

Cortical Thickness

Supplementary Table 2. Linear mixed-effects model fits for each of the 62 DKT atlas regions[1] tested in the cortical thickness analysis.

Region	X^2 Statistic	Df	KS D-statistic	KS p-value	Skew of Residuals	% Residuals Within 2SD of Mean	p-value	pFDR
**lcACC**	0.336	2.000	0.063	0.916	-0.099	95.833	0.845	0.845
**lcMFG**	4.628	2.000	0.130	0.160	-0.144	93.056	0.099	0.157
**lCUN**	6.428	2.000	0.064	0.912	-0.314	93.056	0.040	0.092
**lENT**	7.215	2.000	0.108	0.347	0.002	91.667	0.027	0.072
**lFUS**	1.814	2.000	0.099	0.455	-0.069	93.056	0.404	0.454
**lIPL**	7.265	2.000	0.065	0.899	-0.131	94.444	0.026	0.072
**lITG**	4.262	2.000	0.066	0.891	-0.159	91.667	0.119	0.171

**liCC**	5.671	2.000	0.055	0.971	-0.223	95.833	0.059	0.110
**lLOG**	4.262	2.000	0.055	0.971	0.358	97.222	0.119	0.171
**lLOF**	9.145	2.000	0.098	0.466	-0.185	95.833	0.010	0.049
**lLING**	13.187	2.000	0.102	0.418	-0.323	93.056	0.001	0.032
**lMOF**	10.731	2.000	0.089	0.588	0.234	94.444	0.005	0.040
**lMGH**	1.782	2.000	0.053	0.981	-0.150	95.833	0.410	0.454
**lPARH**	6.002	2.000	0.065	0.905	-0.143	90.278	0.050	0.101
**lparaC**	7.241	2.000	0.113	0.297	-0.395	97.222	0.027	0.072
**lpOPER**	5.972	2.000	0.078	0.743	0.235	97.222	0.050	0.101
**lpORB**	8.450	2.000	0.066	0.888	-0.358	94.444	0.015	0.060
**lpTRI**	5.352	2.000	0.064	0.912	0.263	95.833	0.069	0.119
**lperiCAL**	7.103	2.000	0.076	0.765	-0.443	93.056	0.029	0.072
**lpostC**	6.524	2.000	0.112	0.302	-0.039	93.056	0.038	0.091
**lPCC**	4.527	2.000	0.084	0.660	-0.046	94.444	0.104	0.161
**lPreC**	1.493	2.000	0.094	0.524	0.353	94.444	0.474	0.516
**lPCUN**	7.633	2.000	0.094	0.515	-0.586	94.444	0.022	0.072
**lrACC**	0.499	2.000	0.131	0.156	0.015	93.056	0.779	0.792
**lrMFG**	2.876	2.000	0.117	0.258	-0.129	93.056	0.237	0.283
**lSFG**	4.177	2.000	0.124	0.202	-0.052	91.667	0.124	0.173
**lSPL**	4.637	2.000	0.116	0.264	-0.533	91.667	0.098	0.157
**lSTG**	5.759	2.000	0.052	0.984	-0.276	93.056	0.056	0.109
**lSMAR**	1.817	2.000	0.084	0.658	-0.035	91.667	0.403	0.454
**lTT**	7.083	2.000	0.093	0.528	-0.152	93.056	0.029	0.072
**lINS**	0.862	2.000	0.133	0.141	-0.378	94.444	0.650	0.683
**rcACC**	0.739	2.000	0.085	0.651	-0.090	93.056	0.691	0.714
**rcMFG**	5.561	2.000	0.128	0.170	-0.009	91.667	0.062	0.113

**rCUN**	7.914	2.000	0.053	0.979	0.233	95.833	0.019	0.072
**rENT**	9.203	2.000	0.056	0.967	0.001	93.056	0.010	0.049
**rFUS**	3.947	2.000	0.108	0.343	0.390	95.833	0.139	0.183
**rIPL**	11.526	2.000	0.093	0.526	-0.131	97.222	0.003	0.032
**rITG**	5.155	2.000	0.066	0.894	0.130	95.833	0.076	0.127
**riCC**	6.115	2.000	0.043	0.998	0.164	95.833	0.047	0.101
**rLOG**	9.501	2.000	0.081	0.696	0.410	94.444	0.009	0.049
**rLOF**	9.539	2.000	0.114	0.289	-0.420	95.833	0.008	0.049
**rLING**	10.509	2.000	0.086	0.624	-0.050	94.444	0.005	0.040
**rMOF**	7.373	2.000	0.094	0.511	0.015	93.056	0.025	0.072
**rMGH**	11.719	2.000	0.038	1.000	-0.204	97.222	0.003	0.032
**rPARH**	8.847	2.000	0.081	0.705	-0.647	97.222	0.012	0.053
**rparaC**	3.685	2.000	0.068	0.872	-0.112	94.444	0.158	0.196
**rpOPER**	4.332	2.000	0.092	0.542	-0.437	93.056	0.115	0.171
**rpORB**	13.580	2.000	0.120	0.233	-0.337	95.833	0.001	0.032
**rpTRI**	11.823	2.000	0.071	0.841	-0.264	95.833	0.003	0.032
**rperiCAL**	4.148	2.000	0.091	0.560	0.240	93.056	0.126	0.173
**rpostC**	9.416	2.000	0.087	0.614	-0.094	94.444	0.009	0.049
**rPCC**	5.442	2.000	0.069	0.856	0.233	94.444	0.066	0.117
**rPreC**	7.282	2.000	0.081	0.696	0.129	91.667	0.026	0.072
**rPCUN**	4.111	2.000	0.098	0.469	0.195	95.833	0.128	0.173
**rrACC**	1.034	2.000	0.073	0.813	-0.482	93.056	0.596	0.637
**rrMFG**	6.255	2.000	0.101	0.431	0.067	93.056	0.044	0.097
**rSFG**	3.831	2.000	0.114	0.282	0.006	93.056	0.147	0.190
**rSPL**	3.445	2.000	0.090	0.571	-0.377	93.056	0.179	0.217
**rSTG**	12.848	2.000	0.060	0.944	-0.161	94.444	0.002	0.032

**rSMAR**	3.758	2.000	0.108	0.340	-0.072	95.833	0.153	0.193
**rTT**	7.321	2.000	0.130	0.160	0.626	94.444	0.026	0.072
**rINS**	2.648	2.000	0.071	0.842	0.394	94.444	0.266	0.311

lcACC = left caudal anterior cingulate. lcMFG = left caudal middle frontal. liCC = left isthmus cingulate. lCUN = left cuneus. lENT = left entorhinal. lFUS = left fusiform. lINS = left insula. lIPL = left inferior parietal. lITG = left inferior temporal. lLING = left lingual. lLOG = left lateral occipital. lLOF = left lateral orbitofrontal. lMGH = left middle temporal. lMOF = left medial orbitofrontal. lPARH = left parahippocampal. lPCC = left posterior cingulate. lPCUN = left precuneus. lPreC = left precentral. lrACC = left rostral anterior cingulate. lrMFG = left rostral middle frontal. lSFG = left superior frontal. lSMAR = left supramarginal. lSPL = left superior parietal. lSTG = left superior temporal. lpOPER = left pars opercularis. lpORB = left pars orbitalis. lpTRI = left pars triangularis. lparaC = left paracentral. lperiCAL = left pericalcarine. lpostC = left postcentral. lTT = left transverse temporal. rcACC = right caudal anterior cingulate. rcMFG = right caudal middle frontal. rCUN = right cuneus. rENT = right entorhinal. rFUS = right fusiform. riCC = right isthmus cingulate. rINS = right insula. rIPL = right inferior parietal. rITG = right inferior temporal. rLING = right lingual. rLOG = right lateral occipital. rLOF = right lateral orbitofrontal. rMGH = right middle temporal. rMOF = right medial orbitofrontal. rPARH = right parahippocampal. rPCC = right posterior cingulate. rPCUN = right precuneus. rPreC = right precentral. rrACC = right rostral anterior cingulate. rrMFG = right rostral middle frontal. rSFG = right superior frontal. rSMAR = right supramarginal. rSPL = right superior parietal. rSTG = right superior temporal. rpOPER = right pars opercularis. rpORB = right pars orbitalis. rpTRI = right pars triangularis. rparaC = right paracentral. rperiCAL = right pericalcarine. rpostC = right postcentral. rTT = right transverse temporal.

Note. KS = Kolmogorov-Smirnov test of normality of residuals where ‘D’ statistic indicates test statistic for cumulative distribution function, and ‘p-value’ connotes significant deviation from normality of residuals.

Log-Jacobian

Supplementary Table 3. Linear mixed-effects model fits for each of the subcortical 28 Mindboggle regions[1] tested in the log-jacobian analysis.

Region	X^2 Statistic	Df	KS D-statistic	KS p-value	Skew of Residuals	% Residuals Within 2SD of Mean	p-value	pFDR
**left cerebellum exterior**	0.325	2.000	0.081	0.696	0.084	98.611	0.850	0.989
**left cerebellum white matter**	11.940	2.000	0.044	0.998	0.098	95.833	0.003	0.989
**left thalamus proper**	0.168	2.000	0.061	0.939	-0.060	94.444	0.919	0.097
**left caudate**	11.282	2.000	0.059	0.953	-0.001	93.056	0.004	0.989
**left putamen**	1.771	2.000	0.056	0.966	-0.050	95.833	0.412	0.609
**left pallidum**	2.351	2.000	0.045	0.998	0.094	95.833	0.309	0.553
**Brain stem**	3.116	2.000	0.100	0.435	0.082	97.222	0.211	0.989
**left hippocampus**	3.695	2.000	0.071	0.832	0.116	97.222	0.158	0.804
**left amygdala**	5.542	2.000	0.072	0.826	0.380	97.222	0.063	0.989
**left accumbens area**	1.907	2.000	0.159	0.046	0.736	97.222	0.385	0.989
**left ventral DC**	21.114	2.000	0.065	0.900	-0.309	94.444	0.000	0.097
**left vessel**	6.680	2.000	0.066	0.890	0.212	93.056	0.035	0.989
**right cerebellum exterior**	1.954	2.000	0.056	0.969	-0.146	98.611	0.376	0.356
**right cerebellum white matter**	8.925	2.000	0.089	0.587	-0.335	95.833	0.012	0.989

**right thalamus proper**	7.444	2.000	0.061	0.934	-0.052	95.833	0.024	0.585
**right caudate**	1.874	2.000	0.093	0.530	0.326	93.056	0.392	0.989
**right putamen**	3.905	2.000	0.071	0.839	0.318	95.833	0.142	0.989
**right pallidum**	7.357	2.000	0.081	0.696	0.112	93.056	0.025	0.609
**right hippocampus**	9.148	2.000	0.078	0.745	0.307	95.833	0.010	0.609
**right amygdala**	0.748	2.000	0.060	0.945	0.110	94.444	0.688	0.787
**right accumbens area**	4.482	2.000	0.089	0.593	0.427	93.056	0.106	0.989
**right ventral DC**	14.081	2.000	0.074	0.803	-0.092	94.444	0.001	0.037
**right vessel**	2.914	2.000	0.094	0.519	0.378	95.833	0.233	0.989
**left basal forebrain**	10.370	2.000	0.103	0.408	0.171	94.444	0.006	0.989
**right basal forebrain**	14.606	2.000	0.054	0.976	0.077	94.444	0.001	0.989
**cerebellar vermal lobules I-V**	3.019	2.000	0.066	0.895	-0.220	97.222	0.221	0.989
**cerebellar vermal lobules VI-VII**	2.222	2.000	0.070	0.849	0.278	93.056	0.329	0.901
**cerebellar vermal lobules VIII-X**	3.728	2.000	0.073	0.809	0.186	95.833	0.155	0.609

DC = diencephalon.

Note KS = Kolmogorov-Smirnov test of normality of residuals where 'D' statistic indicates test statistic for cumulative distribution function, and 'p-value' connotes significant deviation from normality of residuals.

Structural Analysis Results

Cortical Thickness Repeated Measures

Supplementary Table 4. Estimated marginal means of cortical thickness estimates for all regions.

Region	Visit	Marginal Mean (mm)	SE	Df	95% CI	
lcACC	Baseline	2.502595	0.057127	23.59921	2.384586	2.620605
	Initial-Post	2.495627	0.057127	23.59921	2.377618	2.613637
	1-Month Post	2.512983	0.057805	24.65741	2.393848	2.632118
lcMFG	Baseline	1.593388	0.040713	26.36137	1.509756	1.67702
	Initial-Post	1.627338	0.040713	26.36137	1.543706	1.71097
	1-Month Post	1.651732	0.0415	28.18079	1.566748	1.736716
lCUN	Baseline	1.55838	0.040494	23.49162	1.474707	1.642052
	Initial-Post	1.601595	0.040494	23.49162	1.517922	1.685267
	1-Month Post	1.604425	0.040962	24.51902	1.519978	1.688873
lENT	Baseline	3.34981	0.106452	21.40735	3.128686	3.570933
	Initial-Post	3.437277	0.106452	21.40735	3.216153	3.6584
	1-Month Post	3.420836	0.106984	21.82626	3.198863	3.642809
lFUS	Baseline	3.403245	0.050972	24.67352	3.298196	3.508293
	Initial-Post	3.436406	0.050972	24.67352	3.331358	3.541455
	1-Month Post	3.43805	0.051731	26.03499	3.331722	3.544379
lIPL	Baseline	1.824167	0.032355	26.31517	1.757699	1.890636
	Initial-Post	1.877307	0.032355	26.31517	1.810839	1.943775
	1-Month Post	1.866621	0.032976	28.1224	1.799086	1.934157
lITG	Baseline	3.065693	0.047353	24.77096	2.968122	3.163264
	Initial-Post	3.112195	0.047353	24.77096	3.014624	3.209766
	1-Month Post	3.116838	0.048071	26.15952	3.018056	3.215621
liCC	Baseline	2.497062	0.042795	23.60456	2.408659	2.585465
	Initial-Post	2.538867	0.042795	23.60456	2.450465	2.62727
	1-Month Post	2.54498	0.043303	24.6643	2.455734	2.634227
lLOG	Baseline	1.618699	0.042479	23.19071	1.530865	1.706534
	Initial-Post	1.657232	0.042479	23.19071	1.569398	1.745067
	1-Month Post	1.652339	0.042932	24.13161	1.563758	1.74092
lLOF	Baseline	2.064837	0.053235	22.7372	1.954641	2.175033
	Initial-Post	2.120376	0.053235	22.7372	2.01018	2.230572

	1-Month Post	2.133578	0.053729	23.54675	2.022573	2.244582
	Baseline	2.233238	0.067246	21.78848	2.0937	2.372776
	Initial-Post	2.314535	0.067246	21.78848	2.174997	2.454073
**lLING**	1-Month Post	2.305902	0.067667	22.32005	2.165686	2.446118
	Baseline	2.118159	0.057001	23.2253	2.000306	2.236012
**lMOF**	Initial-Post	2.189285	0.057001	23.2253	2.071432	2.307138
	1-Month Post	2.202962	0.057615	24.17617	2.084097	2.321828
	Baseline	2.410698	0.058532	25.79086	2.290336	2.53106
**lMGH**	Initial-Post	2.446987	0.058532	25.79086	2.326625	2.567349
	1-Month Post	2.458129	0.059578	27.4583	2.33598	2.580277
	Baseline	2.800769	0.08894	20.83899	2.615721	2.985817
**lPARH**	Initial-Post	2.853397	0.08894	20.83899	2.668349	3.038445
	1-Month Post	2.841178	0.08921	21.08915	2.655704	3.026653
	Baseline	1.468009	0.037206	22.59716	1.390967	1.545051
**lparaC**	Initial-Post	1.507426	0.037206	22.59716	1.430384	1.584468
	1-Month Post	1.503946	0.037535	23.36593	1.426367	1.581526
	Baseline	1.654694	0.043689	22.82334	1.564277	1.745111
**lpOPER**	Initial-Post	1.685306	0.043689	22.82334	1.594889	1.775723
	1-Month Post	1.703916	0.044106	23.65792	1.612815	1.795017
	Baseline	1.676928	0.045776	25.54545	1.582752	1.771104
**lpORB**	Initial-Post	1.743413	0.045776	25.54545	1.649237	1.837589
	1-Month Post	1.752217	0.046565	27.1466	1.656697	1.847737
	Baseline	1.52205	0.045057	25.18929	1.429288	1.614811
**lpTRI**	Initial-Post	1.573298	0.045057	25.18929	1.480536	1.666059
	1-Month Post	1.578795	0.045791	26.6933	1.484789	1.672802
	Baseline	1.340034	0.056239	21.73523	1.223318	1.45675
**lperiCAL**	Initial-Post	1.392824	0.056239	21.73523	1.276108	1.50954
	1-Month Post	1.374348	0.056582	22.25109	1.257082	1.491615
	Baseline	1.08981	0.033675	22.93053	1.020137	1.159483
**lpostC**	Initial-Post	1.119417	0.033675	22.93053	1.049744	1.18909
	1-Month Post	1.128325	0.034007	23.7962	1.058106	1.198544
**lPCC**	Baseline	2.559816	0.069408	20.86943	2.415419	2.704214

	Initial-Post	2.597379	0.069408	20.86943	2.452982	2.741777
	1-Month Post	2.582403	0.069627	21.12864	2.43766	2.727146
	Baseline	1.334146	0.038854	24.2224	1.253995	1.414297
**lPreC**	Initial-Post	1.34638	0.038854	24.2224	1.266229	1.426531
	1-Month Post	1.360919	0.039384	25.45752	1.27988	1.441958
	Baseline	2.123388	0.045544	21.86729	2.028902	2.217875
**lPCUN**	Initial-Post	2.169395	0.045544	21.86729	2.074909	2.263881
	1-Month Post	2.152525	0.045841	22.42209	2.057561	2.24749
	Baseline	3.207763	0.071544	21.31461	3.059112	3.356414
**lrACC**	Initial-Post	3.222655	0.071544	21.31461	3.074004	3.371306
	1-Month Post	3.220059	0.071879	21.70605	3.070874	3.369244
	Baseline	1.811686	0.045381	24.3765	1.7181	1.905272
**lrMFG**	Initial-Post	1.832948	0.045381	24.3765	1.739362	1.926534
	1-Month Post	1.855766	0.046021	25.65495	1.761108	1.950425
	Baseline	1.716315	0.039186	25.12818	1.63563	1.797
**lSFG**	Initial-Post	1.745626	0.039186	25.12818	1.664941	1.826311
	1-Month Post	1.764732	0.039819	26.61542	1.682976	1.846489
	Baseline	1.262193	0.0276	28.74423	1.205722	1.318664
**lSPL**	Initial-Post	1.303118	0.0276	28.74423	1.246647	1.359589
	1-Month Post	1.296445	0.028287	31.16226	1.238764	1.354125
	Baseline	1.898765	0.038525	25.31384	1.819471	1.978058
**lSTG**	Initial-Post	1.942257	0.038525	25.31384	1.862963	2.02155
	1-Month Post	1.951536	0.039166	26.85195	1.871155	2.031918
	Baseline	1.674105	0.036874	26.25458	1.598346	1.749864
**lSMAR**	Initial-Post	1.695867	0.036874	26.25458	1.620108	1.771626
	1-Month Post	1.706238	0.037576	28.04579	1.629274	1.783203
	Baseline	2.055318	0.070112	20.80992	1.909431	2.201205
**lTT**	Initial-Post	2.081618	0.070112	20.80992	1.935731	2.227505
	1-Month Post	2.103445	0.070318	21.05143	1.957233	2.249657
	Baseline	3.310935	0.079204	21.77157	3.146575	3.475294
**lINS**	Initial-Post	3.297011	0.079204	21.77157	3.132651	3.46137
	1-Month Post	3.283164	0.079696	22.29815	3.118013	3.448314

rcACC	Baseline	2.371938	0.067526	22.77225	2.232172	2.511703
	Initial-Post	2.396448	0.067526	22.77225	2.256683	2.536214
	1-Month Post	2.391622	0.06816	23.59198	2.250818	2.532426
rcMFG	Baseline	1.546302	0.049435	23.60174	1.444181	1.648422
	Initial-Post	1.590857	0.049435	23.60174	1.488737	1.692978
	1-Month Post	1.603358	0.050022	24.66067	1.500263	1.706453
rCUN	Baseline	1.595884	0.057616	21.57761	1.476259	1.715508
	Initial-Post	1.617932	0.057616	21.57761	1.498308	1.737557
	1-Month Post	1.653681	0.057937	22.0469	1.533543	1.77382
rENT	Baseline	3.654336	0.081989	22.46754	3.484506	3.824166
	Initial-Post	3.729274	0.081989	22.46754	3.559445	3.899104
	1-Month Post	3.759283	0.082681	23.19847	3.588325	3.930241
rFUS	Baseline	3.450741	0.057762	22.8065	3.331195	3.570288
	Initial-Post	3.499562	0.057762	22.8065	3.380015	3.619108
	1-Month Post	3.489622	0.058311	23.63619	3.369177	3.610068
rIPL	Baseline	1.944861	0.049705	23.25597	1.842101	2.047621
	Initial-Post	2.013705	0.049705	23.25597	1.910945	2.116465
	1-Month Post	2.018154	0.050244	24.21567	1.914504	2.121805
rITG	Baseline	2.871343	0.053696	24.07266	2.760537	2.98215
	Initial-Post	2.926983	0.053696	24.07266	2.816177	3.037789
	1-Month Post	2.929266	0.054407	25.26551	2.817272	3.041259
riCC	Baseline	2.518105	0.050573	22.55685	2.413373	2.622838
	Initial-Post	2.560649	0.050573	22.55685	2.455916	2.665382
	1-Month Post	2.569347	0.051014	23.31386	2.463894	2.674799
rLOG	Baseline	1.618981	0.044167	23.2929	1.527677	1.710285
	Initial-Post	1.674113	0.044167	23.2929	1.582809	1.765417
	1-Month Post	1.679092	0.044652	24.26324	1.586988	1.771195
rLOF	Baseline	2.040489	0.049343	22.61195	1.938317	2.14266
	Initial-Post	2.095656	0.049343	22.61195	1.993485	2.197827
	1-Month Post	2.101292	0.049782	23.38503	1.998404	2.20418
rLING	Baseline	2.160795	0.064209	22.76235	2.027891	2.293698
	Initial-Post	2.238386	0.064209	22.76235	2.105483	2.37129

	1-Month Post	2.245767	0.06481	23.5792	2.11188	2.379655
**rMOF**	Baseline	2.266483	0.061541	23.79025	2.139409	2.393557
	Initial-Post	2.341652	0.061541	23.79025	2.214578	2.468726
	1-Month Post	2.341875	0.062306	24.90296	2.213529	2.470221
**rMGH**	Baseline	2.316801	0.042496	29.37539	2.229935	2.403668
	Initial-Post	2.407846	0.042496	29.37539	2.320979	2.494713
	1-Month Post	2.418694	0.043612	31.94113	2.329852	2.507535
**rPARH**	Baseline	2.717166	0.064948	21.92946	2.582448	2.851884
	Initial-Post	2.782954	0.064948	21.92946	2.648236	2.917672
	1-Month Post	2.778128	0.065384	22.50257	2.642705	2.91355
**rparaC**	Baseline	1.365846	0.04144	23.06819	1.280134	1.451558
	Initial-Post	1.401113	0.04144	23.06819	1.315402	1.486825
	1-Month Post	1.393903	0.041867	23.9737	1.307489	1.480316
**rpOPER**	Baseline	1.697807	0.049981	22.27333	1.594226	1.801388
	Initial-Post	1.736081	0.049981	22.27333	1.632499	1.839662
	1-Month Post	1.733244	0.050373	22.94744	1.629027	1.837461
**rpORB**	Baseline	1.823179	0.04442	24.24196	1.73155	1.914809
	Initial-Post	1.901733	0.04442	24.24196	1.810104	1.993362
	1-Month Post	1.899436	0.045029	25.48259	1.806787	1.992085
**rpTRI**	Baseline	1.48003	0.058119	22.09608	1.35953	1.60053
	Initial-Post	1.545589	0.058119	22.09608	1.425089	1.666089
	1-Month Post	1.551732	0.058541	22.7182	1.430548	1.672915
**rperiCAL**	Baseline	1.463197	0.064946	21.30939	1.328254	1.59814
	Initial-Post	1.485569	0.064946	21.30939	1.350626	1.620512
	1-Month Post	1.506377	0.065249	21.69928	1.370951	1.641803
**rpostC**	Baseline	1.005123	0.030309	24.31327	0.942611	1.067635
	Initial-Post	1.042749	0.030309	24.31327	0.980237	1.105261
	1-Month Post	1.054974	0.030731	25.57396	0.991755	1.118193
**rPCC**	Baseline	2.675002	0.057063	21.43736	2.55648	2.793524
	Initial-Post	2.715572	0.057063	21.43736	2.59705	2.834093
	1-Month Post	2.709577	0.057354	21.86516	2.59059	2.828564
**rPreC**	Baseline	1.304265	0.03496	25.65953	1.232357	1.376173

	Initial-Post	1.352911	0.03496	25.65953	1.281003	1.424819
	1-Month Post	1.357129	0.035573	27.29156	1.284176	1.430083
**rPCUN**	Baseline	2.159893	0.052063	21.79666	2.051863	2.267922
	Initial-Post	2.194952	0.052063	21.79666	2.086923	2.302982
	1-Month Post	2.191631	0.05239	22.33063	2.083075	2.300188
**rrACC**	Baseline	2.988134	0.076036	21.85098	2.830382	3.145885
	Initial-Post	3.002139	0.076036	21.85098	2.844387	3.159891
	1-Month Post	3.017985	0.076528	22.40097	2.859441	3.176529
**rrMFG**	Baseline	1.808856	0.04648	23.7442	1.712871	1.904841
	Initial-Post	1.857111	0.04648	23.7442	1.761126	1.953097
	1-Month Post	1.864665	0.047051	24.84379	1.76773	1.9616
**rSFG**	Baseline	1.718295	0.037225	25.05908	1.641637	1.794952
	Initial-Post	1.750578	0.037225	25.05908	1.67392	1.827236
	1-Month Post	1.759816	0.037819	26.52731	1.682153	1.837479
**rSPL**	Baseline	1.202229	0.038326	24.01036	1.12313	1.281329
	Initial-Post	1.231335	0.038326	24.01036	1.152235	1.310434
	1-Month Post	1.238414	0.038827	25.18559	1.158479	1.318349
**rSTG**	Baseline	1.918431	0.044018	24.27715	1.827636	2.009225
	Initial-Post	1.98357	0.044018	24.27715	1.892775	2.074365
	1-Month Post	2.001822	0.044626	25.52768	1.910009	2.093635
**rSMAR**	Baseline	1.639489	0.045507	24.55307	1.54568	1.733298
	Initial-Post	1.680371	0.045507	24.55307	1.586562	1.77418
	1-Month Post	1.684918	0.04617	25.88095	1.589993	1.779843
**rTT**	Baseline	1.917637	0.080465	20.64068	1.750124	2.085151
	Initial-Post	1.951217	0.080465	20.64068	1.783704	2.11873
	1-Month Post	1.966076	0.080653	20.83179	1.798267	2.133885
**rINS**	Baseline	3.138615	0.072127	21.16634	2.988691	3.288539
	Initial-Post	3.107644	0.072127	21.16634	2.95772	3.257568
	1-Month Post	3.10884	0.072428	21.51379	2.958437	3.259243

CI = confidence interval. SE = standard error. Df = degrees of freedom. lcACC = left caudal anterior cingulate, lcMFG = left caudal middle frontal, lCUN = left cuneus, lENT = left entorhinal, lFUS = left fusiform, lIPL = left inferior parietal, lITG = left inferior temporal, liCC = left isthmus cingulate, lLOG = left lateral occipital, lLOF = left lateral orbitofrontal, lLING = left lingual, lMOF = left medial orbitofrontal, lMGH = left middle temporal, lPARH = left parahippocampal, lparaC = left paracentral, lpOPER = left pars opercularis, lpORB = left pars orbitalis, lpTRI = left pars triangularis, lperiCAL = pericalcarine, lpostC = left postcentral, lPCC = left posterior cingulate, lPreC = left precentral, lPCUN = left precuneus, lrACC = left rostral anterior cingulate, lrMFG = left rostral middle frontal, lSFG = left superior frontal, lSPL = left superior parietal, lSTG = left

superior temporal, lSMAR = left supramarginal, lTT = left transverse temporal, lINS = left insula, rcACC = right caudal anterior cingulate, rcMFG = right caudal middle frontal, rCUN = right cuneus, rENT = right entorhinal, rFUS = right fusiform, rIPL = right inferior parietal, rITG = right inferior temporal, riCC = right isthmus cingulate, rLOG = right lateral occipital, rLOF = right lateral orbitofrontal, rLING = right lingual, rMOF = right medial orbitofrontal, rMGH = right middle temporal, rPARH = right parahippocampal, rparaC = right paracentral, rpOPER = right pars opercularis, rpORB = right pars orbitalis, rpTRI = right pars triangularis, rperiCAL = right pericalcarine, rpostC = right postcentral, rPCC = right posterior cingulate, rPreC = right precentral, rPCUN = right precuneus, rrACC = right rostral anterior cingulate, rrMFG = right rostral middle frontal, rSFG = right superior frontal, rSPL = right superior parietal, rSTG = right superior temporal, rSMAR = right supramarginal, rTT = right transverse temporal, rINS = right insula.

Supplementary Table 5. Post-hoc pairwise comparisons of cortical thickness for regions with significant main effects.

Region	Contrast	Estimate	SE	Df	t-statistic	p-value
**lLOF**	Baseline - Initial-Post	-0.0555	0.0235	45.0006	-2.3617	0.0452
	Baseline - 1-Month Post	-0.0687	0.0247	45.1030	-2.7854	0.0234
	Initial-Post - 1-Month Post	-0.0132	0.0247	45.1030	-0.5349	0.5953
**lLING**	Baseline - Initial-Post	-0.0813	0.0243	45.0002	-3.3399	0.0051
	Baseline - 1-Month Post	-0.0727	0.0255	45.0680	-2.8441	0.0133
	Initial-Post - 1-Month Post	0.0086	0.0255	45.0680	0.3379	0.7370
**lMOF**	Baseline - Initial-Post	-0.0711	0.0271	45.0008	-2.6199	0.0239
	Baseline - 1-Month Post	-0.0848	0.0285	45.1208	-2.9767	0.0140
	Initial-Post - 1-Month Post	-0.0137	0.0285	45.1208	-0.4801	0.6335
**rENT**	Baseline - Initial-Post	-0.0749	0.0345	45.0005	-2.1709	0.0705
	Baseline - 1-Month Post	-0.1049	0.0362	45.0931	-2.8969	0.0174
	Initial-Post - 1-Month Post	-0.0300	0.0362	45.0931	-0.8283	0.4119
**rIPL**	Baseline - Initial-Post	-0.0688	0.0238	45.0008	-2.8956	0.0156
	Baseline - 1-Month Post	-0.0733	0.0249	45.1219	-2.9377	0.0156
	Initial-Post - 1-Month Post	-0.0044	0.0249	45.1219	-0.1783	0.8593
**rLOG**	Baseline - Initial-Post	-0.0551	0.0212	45.0008	-2.5962	0.0294
	Baseline - 1-Month Post	-0.0601	0.0223	45.1232	-2.6975	0.0294
	Initial-Post - 1-Month Post	-0.0050	0.0223	45.1232	-0.2234	0.8242
**rLOF**	Baseline - Initial-Post	-0.0552	0.0213	45.0005	-2.5864	0.0280
	Baseline - 1-Month Post	-0.0608	0.0224	45.0984	-2.7162	0.0280
	Initial-Post - 1-Month Post	-0.0056	0.0224	45.0984	-0.2518	0.8024
**rLING**	Baseline - Initial-Post	-0.0776	0.0285	45.0006	-2.7241	0.0201
	Baseline - 1-Month Post	-0.0850	0.0299	45.1039	-2.8426	0.0201
	Initial-Post - 1-Month Post	-0.0074	0.0299	45.1039	-0.2469	0.8061
**rMGH**	Baseline - Initial-Post	-0.0910	0.0320	45.0059	-2.8409	0.0135
	Baseline - 1-Month Post	-0.1019	0.0336	45.3325	-3.0325	0.0120
	Initial-Post - 1-Month Post	-0.0108	0.0336	45.3325	-0.3228	0.7483
**rpORB**	Baseline - Initial-Post	-0.0786	0.0239	45.0013	-3.2822	0.0060
	Baseline - 1-Month Post	-0.0763	0.0251	45.1572	-3.0367	0.0079
	Initial-Post - 1-Month Post	0.0023	0.0251	45.1572	0.0915	0.9275
**rpTRI**	Baseline - Initial-Post	-0.0656	0.0227	45.0003	-2.8915	0.0127
	Baseline - 1-Month Post	-0.0717	0.0238	45.0795	-3.0131	0.0127
	Initial-Post - 1-Month Post	-0.0061	0.0238	45.0795	-0.2581	0.7975
**rpostC**	Baseline - Initial-Post	-0.0376	0.0165	45.0013	-2.2870	0.0539
	Baseline - 1-Month Post	-0.0499	0.0173	45.1597	-2.8880	0.0178
	Initial-Post - 1-Month Post	-0.0122	0.0173	45.1597	-0.7082	0.4825
**rSTG**	Baseline - Initial-Post	-0.0651	0.0238	45.0013	-2.7364	0.0177
	Baseline - 1-Month Post	-0.0834	0.0250	45.1584	-3.3388	0.0051
	Initial-Post - 1-Month Post	-0.0183	0.0250	45.1584	-0.7308	0.4687

Df = degrees of freedom. lLING = left lingual. lLOF = left lateral orbitofrontal. lMOF = left medial orbitofrontal. rENT = right entorhinal. rIPL = right inferior parietal. rLING = right lingual. rLOF = right lateral orbitofrontal. rLOG = right lateral occipital. rMGH = right middle temporal. rpORB = right pars orbitalis. rpostC = right postcentral. rpTRI = right pars triangularis. rSTG = right superior temporal. SE = standard error. *Note.* p-values are "holm" corrected for 3 pairwise contrasts.

Log-Jacobian Repeated Measures

Supplementary Table 6. ANOVA table of regions with significant main effect of visit for log-jacobian determinant (surviving FDR correction)

Region	X^2 Statistic	Df	*p*value	*p*FDR
**Left Cerebellum White Matter**	11.940	2.000	0.003	0.018*
**Left Caudate**	11.282	2.000	0.004	0.020*
**Left Ventral DC**	21.114	2.000	0.000	0.001**
**Right Cerebellum White Matter**	8.925	2.000	0.012	0.040*
**Right Hippocampus**	9.148	2.000	0.010	0.040*
**Right Ventral DC**	14.081	2.000	0.001	0.008**
**Left Basal Forebrain**	10.370	2.000	0.006	0.026*
**Right Basal Forebrain**	14.606	2.000	0.001	0.008**

Notes. X^2 Statistic: Wald Chi-Square Test (Type II). p-value: uncorrected p-value for main effect of Visit. pFDR: FDR-corrected p-value for 28 regions p<0.05*, p<0.01**, p<0.001***.

Supplementary Table 7. Estimated marginal means of log-jacobian estimates for subcortical regions tested.

Region	Visit	Marginal Mean (A.U.)	SE	Df	95% CI	
	Baseline	-0.02815	0.00412	29.26881	-0.03658	-0.01973
**left cerebellum exterior**	Initial-Post	-0.0264	0.00412	29.26881	-0.03482	-0.01798
	1-Month Post	-0.02743	0.004227	31.80995	-0.03604	-0.01882
**left cerebellum white matter**	Baseline	0.191147	0.00836	30.93371	0.174096	0.208198

	Initial-Post	0.211651	0.00836	30.93371	0.1946	0.228702
	1-Month Post	0.2113	0.008606	33.84246	0.193807	0.228792
**left thalamus proper**	Baseline	-0.00446	0.00372	35.9942	-0.01201	0.00308
	Initial-Post	-0.00388	0.00372	35.9942	-0.01142	0.00367
	1-Month Post	-0.00299	0.003864	39.77735	-0.0108	0.004821
**left caudate**	Baseline	-0.03075	0.005159	29.55802	-0.04129	-0.02021
	Initial-Post	-0.03844	0.005159	29.55802	-0.04898	-0.02789
	1-Month Post	-0.04444	0.005297	32.16557	-0.05523	-0.03365
**left putamen**	Baseline	-0.05721	0.00417	54.53176	-0.06557	-0.04885
	Initial-Post	-0.05438	0.00417	54.53176	-0.06274	-0.04603
	1-Month Post	-0.05012	0.004428	57.7435	-0.05899	-0.04126
**left pallidum**	Baseline	-0.00696	0.007907	34.86267	-0.02302	0.009092
	Initial-Post	0.002559	0.007907	34.86267	-0.0135	0.018615
	1-Month Post	-0.00714	0.008197	38.48439	-0.02372	0.009452
**Brain stem**	Baseline	0.033772	0.003327	43.29633	0.027064	0.040479
	Initial-Post	0.038109	0.003327	43.29633	0.031401	0.044816
	1-Month Post	0.04005	0.00349	47.59987	0.033031	0.047068
**left hippocampus**	Baseline	0.000778	0.004045	29.81302	-0.00749	0.009042
	Initial-Post	0.006518	0.004045	29.81302	-0.00175	0.014781
	1-Month Post	0.005168	0.004155	32.47827	-0.00329	0.013627
**left amygdala**	Baseline	-0.0199	0.007221	36.52892	-0.03454	-0.00526
	Initial-Post	-0.00638	0.007221	36.52892	-0.02102	0.008255
	1-Month Post	-0.00538	0.007506	40.38113	-0.02054	0.009789
**left accumbens area**	Baseline	-0.05475	0.00533	41.8579	-0.06551	-0.044

	Initial-Post	-0.05877	0.00533	41.8579	-0.06953	-0.04801
	1-Month Post	-0.0508	0.005582	46.13302	-0.06203	-0.03956
**left ventral DC**	Baseline	-0.00239	0.003221	45.41503	-0.00888	0.004096
	Initial-Post	0.012105	0.003221	45.41503	0.005619	0.018592
	1-Month Post	0.011513	0.003388	49.69201	0.004707	0.018319
**left vessel**	Baseline	-0.07571	0.010197	45.06613	-0.09625	-0.05518
	Initial-Post	-0.06119	0.010197	45.06613	-0.08172	-0.04065
	1-Month Post	-0.04572	0.01072	49.35313	-0.06726	-0.02419
**right cerebellum exterior**	Baseline	-0.01631	0.00354	34.21689	-0.02351	-0.00912
	Initial-Post	-0.01199	0.00354	34.21689	-0.01918	-0.0048
	1-Month Post	-0.01349	0.003666	37.73737	-0.02091	-0.00606
**right cerebellum white matter**	Baseline	0.177894	0.008381	31.45558	0.160811	0.194977
	Initial-Post	0.197412	0.008381	31.45558	0.180329	0.214495
	1-Month Post	0.193228	0.008636	34.47196	0.175686	0.210771
**right thalamus proper**	Baseline	-0.00177	0.0038	28.3539	-0.00955	0.006006
	Initial-Post	0.001716	0.0038	28.3539	-0.00606	0.009495
	1-Month Post	0.006047	0.003891	30.6782	-0.00189	0.013986
**right caudate**	Baseline	-0.02914	0.00682	39.15338	-0.04293	-0.01535
	Initial-Post	-0.02489	0.00682	39.15338	-0.03868	-0.01109
	1-Month Post	-0.03455	0.007116	43.27525	-0.04889	-0.0202
**right putamen**	Baseline	-0.06399	0.004618	48.62384	-0.07327	-0.05471
	Initial-Post	-0.05927	0.004618	48.62384	-0.06855	-0.04998
	1-Month Post	-0.0531	0.004875	52.70314	-0.06288	-0.04332
**right pallidum**	Baseline	-0.03525	0.010482	24.59279	-0.05685	-0.01364

	Initial-Post	-0.02246	0.010482	24.59279	-0.04407	-0.00086
	1-Month Post	-0.02019	0.010636	25.93176	-0.04205	0.001681
**right hippocampus**	Baseline	0.001462	0.003223	31.18914	-0.00511	0.008034
	Initial-Post	0.008781	0.003223	31.18914	0.002209	0.015352
	1-Month Post	0.002423	0.003319	34.15103	-0.00432	0.009168
**right amygdala**	Baseline	0.001489	0.008977	28.38924	-0.01689	0.019866
	Initial-Post	0.006643	0.008977	28.38924	-0.01173	0.02502
	1-Month Post	0.002057	0.009193	30.7221	-0.0167	0.020814
**right accumbens area**	Baseline	-0.03241	0.005668	48.44947	-0.0438	-0.02102
	Initial-Post	-0.03085	0.005668	48.44947	-0.04224	-0.01945
	1-Month Post	-0.01917	0.005981	52.54442	-0.03116	-0.00717
**right ventral DC**	Baseline	0.004159	0.003353	40.29602	-0.00262	0.010933
	Initial-Post	0.016249	0.003353	40.29602	0.009475	0.023024
	1-Month Post	0.013804	0.003504	44.49835	0.006745	0.020863
**right vessel**	Baseline	-0.06461	0.011533	40.90756	-0.08791	-0.04132
	Initial-Post	-0.0614	0.011533	40.90756	-0.0847	-0.03811
	1-Month Post	-0.04478	0.012062	45.14354	-0.06907	-0.02049
**left basal forebrain**	Baseline	-0.06512	0.012455	25.61633	-0.09074	-0.0395
	Initial-Post	-0.04226	0.012455	25.61633	-0.06788	-0.01663
	1-Month Post	-0.04574	0.012672	27.23669	-0.07173	-0.01975
**right basal forebrain**	Baseline	-0.01612	0.011423	27.8539	-0.03953	0.007283
	Initial-Post	0.014483	0.011423	27.8539	-0.00892	0.037888
	1-Month Post	0.000461	0.011685	30.05555	-0.0234	0.024323
**cerebellar vermal lobules I-V**	Baseline	-0.03747	0.005288	34.15067	-0.04821	-0.02672

	Initial-Post	-0.04115	0.005288	34.15067	-0.05189	-0.0304
	1-Month Post	-0.03264	0.005475	37.6604	-0.04372	-0.02155
**cerebellar vermal lobules VI-VII**	Baseline	-0.0752	0.009777	25.90295	-0.0953	-0.0551
	Initial-Post	-0.07762	0.009777	25.90295	-0.09772	-0.05751
	1-Month Post	-0.06835	0.009955	27.60048	-0.08875	-0.04794
**cerebellar vermal lobules VIII-X**	Baseline	-0.02804	0.007593	25.00883	-0.04368	-0.0124
	Initial-Post	-0.03468	0.007593	25.00883	-0.05032	-0.01904
	1-Month Post	-0.03624	0.007713	26.46321	-0.05209	-0.0204

CI = confidence interval. DC= diencephalon. Df = degrees of freedom. SE = standard error.

Supplementary Table 8. Post-hoc pairwise comparisons of log-jacobian estimates for subcortical regions with significant main effects.

Region	Contrast	Estimate	SE	Df	*T* statistic	p-value
**Left Cerebellum White Matter**	Baseline - Initial-Post	-0.0205	0.0067	45.0079	-3.0601	0.0112
	Baseline - 1-Month Post	-0.0202	0.007	45.3838	-2.8693	0.0125
	Initial-Post - 1-Month Post	0.0004	0.007	45.3838	0.05	0.9603
**Left Caudate**	Baseline - Initial-Post	0.0077	0.0039	45.0062	1.9609	0.1122
	Baseline - 1-Month Post	0.0137	0.0041	45.3386	3.3305	0.0052
	Initial-Post - 1-Month Post	0.006	0.0041	45.3386	1.4602	0.1511
**Left Ventral DC**	Baseline - Initial-Post	-0.0145	0.0035	45.0411	-4.1089	0.0005
	Baseline - 1-Month Post	-0.0139	0.0037	45.8549	-3.7681	0.0009
	Initial-Post - 1-Month Post	0.0006	0.0037	45.8549	0.1605	0.8732
**Right Cerebellum White Matter**	Baseline - Initial-Post	-0.0195	0.0068	45.0087	-2.8533	0.0196
	Baseline - 1-Month Post	-0.0153	0.0072	45.4008	-2.1387	0.0758
	Initial-Post - 1-Month Post	0.0042	0.0072	45.4008	0.5835	0.5624
**Right Hippocampus**	Baseline - Initial-Post	-0.0073	0.0026	45.0083	-2.8077	0.0221
	Baseline - 1-Month Post	-0.001	0.0027	45.3921	-0.3517	0.7267
	Initial-Post - 1-Month Post	0.0064	0.0027	45.3921	2.327	0.049
**Right Ventral DC**	Baseline - Initial-Post	-0.0121	0.0034	45.0260	-3.5696	0.0026
	Baseline - 1-Month Post	-0.0096	0.0035	45.6853	-2.7202	0.0184
	Initial-Post - 1-Month Post	0.0035	0.0247	45.6853	0.6898	0.4938

**Left Basal Forebrain**	Baseline - Initial-Post	-0.0229	0.0076	45.0022	-3.0131	0.0127
	Baseline - 1-Month Post	-0.0194	0.008	45.2054	-2.4345	0.0378
	Initial-Post - 1-Month Post	0.0035	0.008	45.2054	0.4378	0.6636
**Right Basal Forebrain**	Baseline - Initial-Post	-0.0306	0.008	45.0042	-3.8179	0.0012
	Baseline - 1-Month Post	-0.0166	0.0084	45.2818	-1.9727	0.1093
	Initial-Post - 1-Month Post	0.014	0.0084	45.2818	1.668	0.1093

CI = confidence interval. Df = degrees of freedom. SE = standard error.
Note. p-values are "holm" corrected for 3 pairwise contrasts.

Repeated Measures Normative Modeling for Cortical Thickness

Supplementary Table 9. Anova Table of Whole Brain and Targeted normative percentile changes

Region	X^2 Statistic	Df	p-value	pFDR
**Whole Brain**	6.7235	2	0.03467	0.03467
**Targeted**	13.3017	2	0.001293	0.00259

Supplementary Table 10. Estimated Marginal Means of normative percentile estimates

Region	Visit	Marginal Mean (Percentile)	SE	Df	95% CI	
**Whole Brain**	Baseline	46.4	3.74	28.3	38.8	54.1
	Initial-Post	51.3	3.74	28.3	43.7	59.0
	1-Month Post	52.0	3.80	30.1	44.2	59.7
**Targeted**	Baseline	43.9	3.76	29.8	36.3	51.6
	Initial-Post	51.4	3.76	29.8	43.7	59.1
	1-Month Post	52.6	3.84	32.0	44.8	60.5

Supplementary Table 11. Post-hoc pairwise comparisons of normative percentile estimates. *Note.* p-values are "holm" corrected for 3 pairwise contrasts.

Region	Contrast	Estimate	SE	Df	t-statistic	p-value
**Whole Brain**	Baseline - Initial-Post	-4.921	2.30	45.0	-2.144	0.0777
	Baseline - 1-Month Post	-5.546	2.41	45.2	-2.303	0.0777

	Initial-Post - 1-Month Post	-0.624	2.41	45.2	-0.259	0.7965
**Targeted**	Baseline - Initial-Post	-7.49	2.53	45.0	-2.964	0.0097
	Baseline - 1-Month Post	-8.70	2.65	45.3	-3.280	0.0060
	Initial-Post - 1-Month Post	-1.20	2.65	45.3	-0.454	0.6521

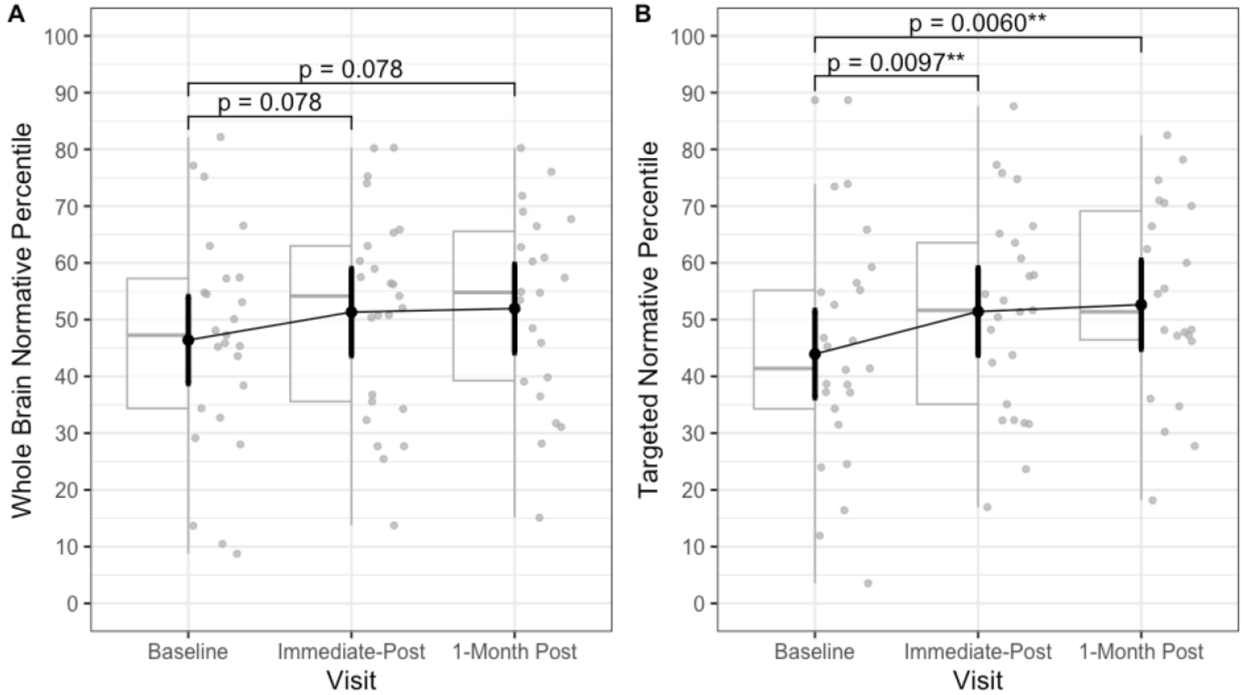

Supplementary Figure 4. Normative cortical thickness estimates across study visits. Box-jitter plots of group-level normative percentile rank in SOV participants at baseline, initial-post, and 1-month post MISTIC for the Whole Brain (A) and A-priori *Targeted* ROIs (B). *Note.* Box-jitter light vertical whiskers represent the 1.5 times the interquartile range (IQR) extending from the box edges. Boxes represent medians (solid horizontal lines) and IQR (box edges), solid circles represent model estimated marginal means, thick error-bars represent model-based standard errors, p-values are “Holm” Bonferroni corrected.

Supplementary Table 12. Correlations between morphological changes and changes in the WHODAS-2.0.

	Initial Post - Baseline		1-Month Post - Baseline	
**Region**	**rho**	**p-value**	**rho**	**p-value**
	Cortical Thickness			
**lLING**	**-0.196**	**0.347**	**0.127**	**0.584**
**lLOF**	**0.085**	**0.685**	**0.004**	**0.987**
**lMOF**	**0.036**	**0.864**	**0.276**	**0.227**
**rENT**	**0.045**	**0.831**	**0.084**	**0.718**
**rIPL**	**-0.135**	**0.519**	**0.084**	**0.716**
**rLING**	**-0.113**	**0.589**	**0.083**	**0.72**

**rLOF**	**0.016**	**0.94**	**0.047**	**0.84**
**rLOG**	**-0.049**	**0.817**	**0.088**	**0.703**
**rMGH**	**-0.023**	**0.913**	**0.143**	**0.536**
**rpORB**	**0.055**	**0.793**	**0.152**	**0.511**
**rpostC**	**0.028**	**0.893**	**-0.025**	**0.913**
**rpTRI**	**0.072**	**0.733**	**-0.254**	**0.266**
**rSTG**	**-0.058**	**0.784**	**0.107**	**0.646**
	Normative Modeling			
**Whole Brain**	**-0.105**	**0.619**	**0.038**	**0.869**
**Targeted**	**-0.097**	**0.645**	**0.038**	**0.869**
	Log-Jacobian			
**Left Basal Forebrain**	**-0.16**	**0.445**	**0.314**	**0.165**
**Left Caudate**	**0.103**	**0.625**	**-0.246**	**0.282**
**Left Cerebellum White Matter**	**-0.345**	**0.092**	**0.022**	**0.924**
**Left Ventral DC**	**-0.046**	**0.828**	**0.094**	**0.687**
**Right Basal Forebrain**	**-0.104**	**0.621**	**0.122**	**0.6**
**Right Cerebellum White Matter**	**-0.225**	**0.279**	**-0.019**	**0.933**
**Right Hippocampus**	**0.012**	**0.955**	**0.024**	**0.918**
**Right Ventral DC**	**-0.332**	**0.105**	**-0.005**	**0.984**
	Predicted Brain Age			
**Predicted Brain Age**	**0.084**	**0.690**	**-0.131**	**0.573**

Where significant regional effects were detected, we conducted exploratory Spearman correlations between the raw changes in regional estimates and the changes in the total score on the WHODAS-2.0. This test was selected because it was the primary outcome measure for the prior study from our group.[1] Correlations between changes from baseline to the initial post time point in WHODAS-2.0 and brain structure were tested, as were correlations between changes from baseline to the 1 month time point in both WHODAS-2.0 and brain structure. No significant correlations were found.

Note: Values in the table above reflect Spearman's rho correlations between changes in scores on the WHODAS-2.0 and changes in morphological variables over the same periods of time. Morphological variables tested are listed in the left column. Time periods over which changes were considered are listed across the top. Values are provided for rho and p value (significance). No correlations were significant, and so none were corrected for multiple comparisons.

References

1. Klein, A., Ghosh, S.S., Bao, F.S., Giard, J., Häme, Y., Stavsky, E., Lee, N., Rossa, B., Reuter, M., Neto, E.C., et al. (2017). Mindboggling morphometry of human brains. PLOS Comput. Biol. *13*, e1005350. https://doi.org/10.1371/journal.pcbi.1005350.
